# Modulation of miRNA Expression by Dietary Polyphenols in apoE Deficient Mice: A New Mechanism of the Action of Polyphenols

**DOI:** 10.1371/journal.pone.0029837

**Published:** 2012-01-10

**Authors:** Dragan Milenkovic, Christiane Deval, Erwan Gouranton, Jean-François Landrier, Augustin Scalbert, Christine Morand, Andrzej Mazur

**Affiliations:** 1 INRA, UMR1019, UNH, CRNH Auvergne, Clermont-Ferrand, Clermont Université, Université d'Auvergne, Unité de Nutrition Humaine, Clermont-Ferrand, France; 2 INRA, UMR1260, Nutriments Lipidiques et Prévention des Maladies Métaboliques, Marseille, France; 3 Université d'Aix-Marseille 1, Marseille, France; 4 Université d'Aix-Marseille 2, Marseille, France; University of Texas MD Anderson Cancer Center, United States of America

## Abstract

**Background:**

Polyphenols are the most abundant antioxidants in the human diet and are widespread constituents of fruits and beverages, such as tea, coffee or wine. Epidemiological, clinical and animal studies support a role of polyphenols in the prevention of various diseases, such as cardiovascular diseases, cancers or neurodegenerative diseases. Recent findings suggest that polyphenols could interact with cellular signaling cascades regulating the activity of transcription factors and consequently affecting the expression of genes. However, the impact of polyphenol on the expression of microRNA, small non-coding RNAs, has not yet been studied. The aim of this study was to investigate the impact of dietary supplementation with polyphenols at nutritional doses on miRNA expression in the livers of apolipoprotein E-deficient mice (apoE−/−) jointly with mRNA expression profiling.

**Methodology/Principal Findings:**

Using microarrays, we measured the global miRNA expression in the livers of wild-type (C57B6/J) mice or apoE−/− mice fed diets supplemented with one of nine different polyphenols or a control diet. This analysis revealed that knock-out of the apoE gene induced significant modulation in the expression of miRNA. Moreover, changes in miRNA expression were observed after polyphenol supplementation, and five miRNAs (mmu-miR-291b-5p, mmu-miR-296-5p, mmu-miR-30c-1*, mmu-miR-467b* and mmu-miR-374*) were identified as being commonly modulated by these polyphenols. We also observed that these polyphenols counteracted the modulation of miRNA expression induced by apoE mutation. Pathway analyses on these five miRNA-target genes revealed common pathways, some of which were also identified from a pathway analysis on mRNA profiles.

**Conclusion:**

This *in vivo* study demonstrated for the first time that polyphenols at nutritional doses modulate the expression of miRNA in the liver. Even if structurally different, all polyphenols induced a similar miRNA expression profile. Common pathways were identified from both miRNA-target and mRNA analysis, revealing cellular functions that could be regulated by polyphenols at both the miRNA and mRNA level.

## Introduction

MicroRNAs (miRNAs) are endogenous, noncoding, single-stranded RNAs of 22 nucleotides and constitute a class of gene regulators [1]. MiRNAs are initially transcribed by RNA polymerase II (Pol II) in the nucleus to form large pri-miRNA transcripts. The pri-miRNAs are processed by the RNase III enzymes, Drosha and Dicer, to generate 18- to 24-nucleotide mature miRNAs [2]. More than 700 miRNAs have been cloned and sequenced in the human [3] and it is believed that miRNAs control the post-transcriptional regulation of 30% of mammalian genes [4]. The mature miRNAs negatively regulate gene expression depending on the degree of complementarity between the miRNA and its target; miRNAs that bind to the 3′ UTR of mRNA with imperfect complementarity block protein translation, while miRNAs that bind to mRNA with perfect complementarity induce targeted mRNA cleavage. Through modifying the availability of mRNAs and in consequence protein synthesis, miRNAs control many cellular processes, such as cell differentiation, growth, proliferation and apoptosis [5]. Changes in miRNAs expression profiles are being extensively studied in human diseases, such as cancer, skeletal muscle diseases or cardiovascular diseases [6], [7], [8], [9], [10], [11].

A wealth of evidence points out that diet is one of the most important modifiable determinants for developing a number of chronic diseases. In particular, consumption of plant-based foods protects against the development of diseases, such as cancers or cardiovascular diseases. Such effects have been ascribed in part to non-nutritive bioactive secondary metabolites. Polyphenols are ubiquitous secondary metabolites found in fruits, vegetables, wholegrain cereals and beverages, such as tea, coffee and wine. Over 500 polyphenols have been identified in various foods [12]. These compounds may be classified into different groups depending on their chemical structures: phenolic acids, flavonoids, stilbenes, lignans and a specific group of curcuminoids. Certain polyphenols, such as quercetin, are found in many foods whereas others are specific to particular foods. It has been estimated that humans consume about 1 g of polyphenol per day with phenolic acids as major class consumed [13]. Epidemiological, clinical or animal studies have suggested an inverse association between the consumption of polyphenol and polyphenol-rich foods or beverages and the prevention of diseases [14], [15], [16], [17]. Growing evidence suggests that the cellular effect mediating their beneficial health effects are related to their capacity to interact with cellular signaling cascades that regulate transcription factors and consequently the expression of genes and proteins rather than to their direct antioxidant capacity [18], [19], [20]. Gene and protein expression modulation results in modification of different cellular processes such as apoptosis, cell cycle or migration, processes that can be regulated by miRNAs. Certain studies assert that some nutrients in foods, such as amino acid or fatty acids, can modulate miRNA expression [21], [22]. It has also been described that retinoic acid, folate or curcumin alter the expression of miRNA, an effect that may contribute to the cancer-protective effects of these nutrients [23]. Regarding the important role of polyphenols in the prevention of disease development, we could hypothesize that these micronutrients could also exert their preventive effect through modulation of expression of miRNA. However, the impact of polyphenol consumption on expression of these non-coding RNAs has not been yet studied.

The objective of the present work was to investigate the impact of nine dietary polyphenols on the expression of miRNA in the liver of apolipoprotein E-deficient mice. This animal model shows altered lipid metabolism in the liver and spontaneously develops atherosclerotic lesions. We measured both miRNA and mRNA expression in the liver with microarrays. For the first time, we show that low nutritional doses of polyphenols supplemented in the diet can modulate expression of miRNA *in vivo* with five miRNAs being commonly modulated by all nine polyphenols tested. The biological pathways modulated by these five miRNAs were identified and 30 of them were found to be also linked to modulated mRNA.

## Materials and Methods

### Animals and diets

**Figure 1:**
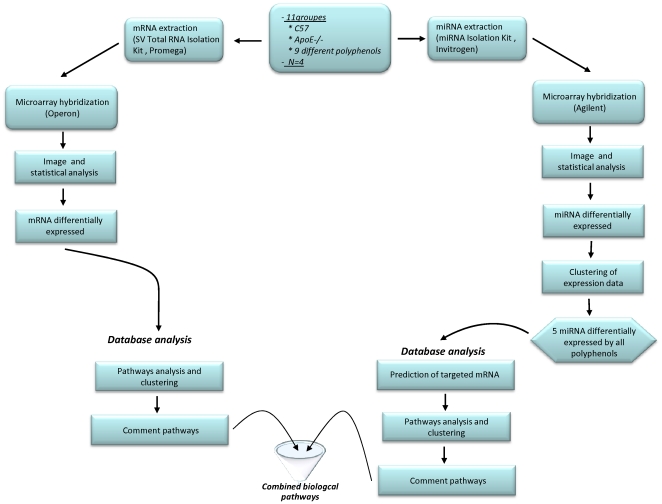
The workflow of experimental approaches adopted for miRNA and mRNA expression analysis.

Wild-type mice and homozygous apoE-deficient mice, which have the same genetic background (C57BL/6), originated from Jackson Laboratories (Charles River Laboratories, L'Arbresle, France) and interbred to obtain the males, were used for the present study. Mice were individually housed in wire-bottomed cages in a temperature-controlled room (22±0.8°C) with a 12 h light–dark cycle and a relative humidity of 55±10%. The mice had free access to food and water. All animals were maintained and handled according to the recommendations of the Institutional Ethics Committee of the INRA, in accordance with decree No 87-848 and this animal experiment was approved by the Ethics Committee in Animal Experiment “CEMEAAuvergne” under registration number CE28-10. All animals were fed semi-synthetic chow diet (Table S1). At eight weeks of age, mice were divided into 10 groups with six mice per group and fed either the control-diet or the same diet supplemented with quercetin, hesperidin, naringenin, anthocyanin, catechin and curcumin at 0.006% (w/w) corresponding to an equivalent intake in humans of 30 mg/day, while proanthocyanin, caffeic acid and ferulic acid were added at 0.06% (w/w) corresponding to an equivalent intake in humans of 300 mg/day. The chemical structures of these polyphenols are presented in Figure S1. After two weeks of supplementation, mice were sacrificed under pentobarbital anesthesia. The organs were washed with physiological saline solution maintained at 37°C by direct injection in the heart's left ventricle. Livers were collected, immediately frozen in liquid nitrogen and stored at −80°C until the time of analysis. Figure 1 summarizes the workflow of the different approaches used in the work.

### MicroRNA microarray analyses

Livers stored at −80°C were grounded in liquid nitrogen and the resulting powder was used for total RNA extraction including miRNA from 4 livers per group using the Qiagen miRNeasy mini kit (Qiagen, France) according to manufacturer's instructions. MiRNAs were labeled using miRNA labeling and hybridization kit from Agilent technologies (Agilent, USA). Briefly, 100 ng of each total RNA sample were treated with calf intestinal phosphatase for 30 min at 37°C before denaturing the samples using pure DMSO at 100°C for 5 min and rapidly transfer in an ice water bath to prevent RNA reannealing. RNA samples were labeled with pCp-Cy3 using T4 RNA ligase by incubation at 16°C for 2 h. After purification with microBioSpin columns, labeled samples were hybridized to Agilent mouse miRNA microarrays, which contain probes for 567 mouse micro RNAs of the Sanger database. Hybridizations were performed for 24 h at 55°C. After hybridization of the miRNA microarray, the microarray was washed in GE Wash Buffer 1 (Agilent, Santa Clara, CA, USA) and GE Wash Buffer 2 (Agilent, Santa Clara, CA, USA) for 5 min. The slides were then scanned with Aligent Microarray Scanner (Agilent, Santa Clara, CA, USA). The scanned images were analyzed by Feature Extraction Software (Agilent, Santa Clara, CA, USA); normalization software (Genespring GX10, Agilent, Santa Clara, CA, USA) was used to quantify the signal and background intensity for each feature and to substantially normalize the data by the 75th percentile method. The standard of statistical significance was the corrected ratios of hybridization signal intensity between polyphenol-supplemented samples and control-diet tissues. miRNAs were considered significantly differently expressed if their ratios were more than 1.5 or less than 0.75.

### Biological function analysis of miRNA: target genes and pathway prediction

Prediction of potential target genes of the regulated miRNA of interest were based on sequence comparison searched using the miRBase database (Sanger institute: http:/microrna.sanger.ac.uk/) (Griffiths-Jones et al., 2008 nucleic acid research). The target gene list was submitted to the KEGG database (Kyoto Encyclopedia of Genes and Genomes; http://www.genome.jp/kegg/tool/color_pathway.html) to place the genes into functional pathways.

### mRNA microarray analyses

#### RNA extraction and fluorescent labeling

The same grounded liver samples as used for miRNA extraction were homogenized in buffer for total RNA extraction using the SV Total RNA Isolation System (Promega, Madison, WI, USA) as recommended by the manufacturer. Total RNAs were extracted from 4 livers per group. The quality of total RNA was monitored by 1% agarose gel electrophoresis. cDNA were labeled using ChipShot™ Direct Labeling System kit (Promega) as recommended by the manufacturer. cDNAs were obtained from 5 μg of total RNA with 1 μL of random primer and 1 µL of oligo(dT), and labeling was performed with Cy™3- or Cy™5-dCTP (GE Healthcare). The labeled cDNA was purified by application to an equilibrated filter cartridge using the ChipShot™ Membrane Clean-Up System (Promega). Quantities and labeling efficiencies of labeled cDNAs were determined by measuring the absorbencies at 260, 550 and 650 nm using a ND-1000 spectrophotometer (Nanodrop).

#### Hybridization

Hybridization was carried out on the Operon mouse microarray (OpArrays™). Array-Ready OligoSet Mouse Genome version 4.0 contains 35,852 longmer probes representing approximately 24,000 genes. Hybridization was carried out in a Ventana hybridization system (Ventana Medical Systems, S.A, Illkirch, France) at 42°C for eight hours. Slides were subsequently washed twice in 2× saline sodium citrate (SSC) and 0.1× SSC at room temperature. The buffer remaining on the slide was removed by rapid centrifugation (4000 *g*, 15 sec). The fluorescence intensity was scanned using the Agilent Micro Array Scanner G2505B (Agilent Technologies, Inc., Santa Clara, CA, USA).

#### Image and data analysis

Image and statistical analysis have been performed as previously described [19]. Briefly, the signal and background intensity values for each spot in both channels were obtained using ImaGene 6.0 software (Biodiscovery, Inc, Proteigene, Saint Marcel, France). Data were filtered using the ImaGene “empty spot” option, which automatically flags low-expressed and missing spots to remove them from the analyses. After base-2 logarithm transformation, data were corrected for systemic dye bias by Lowess normalization using GeneSight 4.1 software (BioDiscovery, Inc, Proteigene). Ratios were then filtered in accordance with their variability among the four comparisons, and genes with high variability were removed from the analysis. Statistical analyses were performed using the free R 2.1 software (http://www.r-project.org). The log ratio between experimental and control samples was analyzed with Student's t test to detect differentially expressed genes and probability values were adjusted using the Bonferroni correction for multiple testing at 1% to eliminate false positives. Genes selected by these criteria are referred to as “differentially expressed genes”.

#### Biological function analysis of differentially expressed mRNA: pathway prediction

As for the target gene lists of miRNA, differentially expressed mRNAs were submitted to the KEGG database (Kyoto Encyclopedia of Genes and Genomes; http://www.genome.jp/kegg/tool/color_pathway.html) in order to place the genes into functional pathways.

### Quantitative real-time PCR

The expression level of 8 potential target genes of the 5 commonly regulated miRNA was measured using quantitative reverse transcription-polymerase chain reaction (qRT-PCR). High-Capacity cDNA Reverse Transcription Kit (AppliedBiosystems, CA, USA) was used to reverse transcribe RNA to cDNA as recommended by the manufacturer. The primers were identified using Primer Express software (AppliedBiosystem, CA, USA) to amplify fragments of 80–180 bp in length and have been synthesized by Eurofins MWG Operon (Ebersberg, Germany). The primers were: Atg5 (5′-CTGGATGGGACTGCAGAATG-3′ and 5′- CGGAACAGCTTCTGGATGAA-3′); Itga6 (5′-CGAGGTCACCTTTGACACCA-3′ and 5′-CTGCGAAGGCTTAGCGACTC-3′); Nckap1 (5′-GCTGCTGGGTTACCATGTGA-3′ and 5′-GGCCAGTGTAGGCAAGGAAA-3′); Sorbs1 (5′-CAGACAGGAGTCAGCCCTCA-3′ and 5′-AAGTGCCAACAAACCATCCA-3′); Akt1 (5′-AGGTTGCCCACACGCTTACT-3′ and GCTCTCGAGACAGGTGGAAGA-3′); Gapdh (5′-GACTCCACTCACGGCAAATTCA-3′ and 5′-TCGCTCCTGGAAGATGGTGAT-3′) Apc (5′-GACGGCAGCTGGAGTATGAA-3′) and 5′-CACGCGCAGTATGTCCTTTT-3′); Lmo7 (5′-TCAGGGATGCTTCCAACTGA-3′ and 5′-GAGGGGGCTTGATCCTTCTT-3′); Msn (5′-TGGCTTCAGAAATGGCAGAG-3′ and 5′-TCATGGCAGTCTTCAGCTCAG-3′). The qRT-PCR was carried out on Applied Biosystems 7900HT Real-Time PCR System (AppliedBiosystem, CA, USA) using the Power SYBR®Green PCR Master Mix kit (Applied Biosystems, Warrington, UK). PCR conditions were: initial denaturation at 95°C for 10 min, a two-step cycling conditions: 15 sec denaturation at 95°C and annealing/extension at 60°C for 30 s, cycled 40 times. PCR reactions were performed in triplicates. The expression levels were calculated using the Δ Δ CT method.

### Unsupervised modeling

The clustering analysis of the miRNA profiles were performed on miRNA or mRNA identified in at least one of the studied conditions. Unsupervised modeling was subsequently performed using hierarchical clustering with the Euclidean distance for calculating the similarity between genes and the ward's distance for the similarity between conditions using average linkage. Permutmatrix version 1.9.3 was used for hierarchical clustering [24].

## Results

In this study, Agilent mouse miRNA microarrays containing probes for 567 miRNA were used to assess the impact of different dietary polyphenols on miRNA expression in an apolipoprotein E knock-out mice model. Nine different polyphenols were tested: phenolic acids (caffeic acid, ferulic acid), flavonoids (quercetin, hesperidin, naringenin, anthocyanin, catechin, proanthocyanin) and curcumin supplemented in the experimental diet for two weeks. We also analyzed the impact of the knock-out of apoE gene in mice on the miRNA expression profile by comparison to wild mice.

### Modulation of expression of miRNA

Mutagenesis of the apolipoprotein E genes induced significant modulation in expression of 119 miRNA, among which 27 were observed as down-regulated and 92 as up-regulated. This condition resulted in the highest down-regulation of miRNA expression with an observed fold-change of −9.72 for miR-801. Polyphenol supplementation also modulated miRNA expression. The number of modulated miRNAs varied according to the tested molecules; only 29 miRNAs were differentially expressed for caffeic acid, whereas hesperidin modified the expression of as much as 97 miRNAs (Table 1). The average fold-change for down-regulated miRNAs was observed to be −2 while the average fold-change for up-regulated ones was 2.24 (fold-changes are presented in Table S2). The highest down regulation observed was −5.89 for mmu-miR-697 in mice supplemented with catechin, while the highest up-regulation was 9.47 for mmu-miR-133b in mice after proanthocyanin supplementation.

**Table 1:** Number of total as well as up- and down-regulated miRNA identified using miRNA microarrays for the different conditions tested.

	Total	down	Up
ApoE−/− *vs* C57BL/6	119	27	92
Quercetin	47	22	25
Hesperidin	97	53	44
Narangin	69	33	36
Anthocyanin	45	30	15
Catechin	80	36	44
Proanthocyanin	55	37	18
Caffeic acid	29	18	11
Ferulic acid	39	17	22
Curcumin	66	55	11

These conditions are: apoE knock-out mutagenesis versus C57BL/6 mice and apoE−/− mice supplemented with one of nine different polyphenols (quercetin, hesperidin, naringenin, anthocyanin, catechin, proanthocyanin, caffeic acid, ferulic acid, curcumin) versus apoE−/− mice.

### Comparison of expression of miRNA

**Figure 2:**
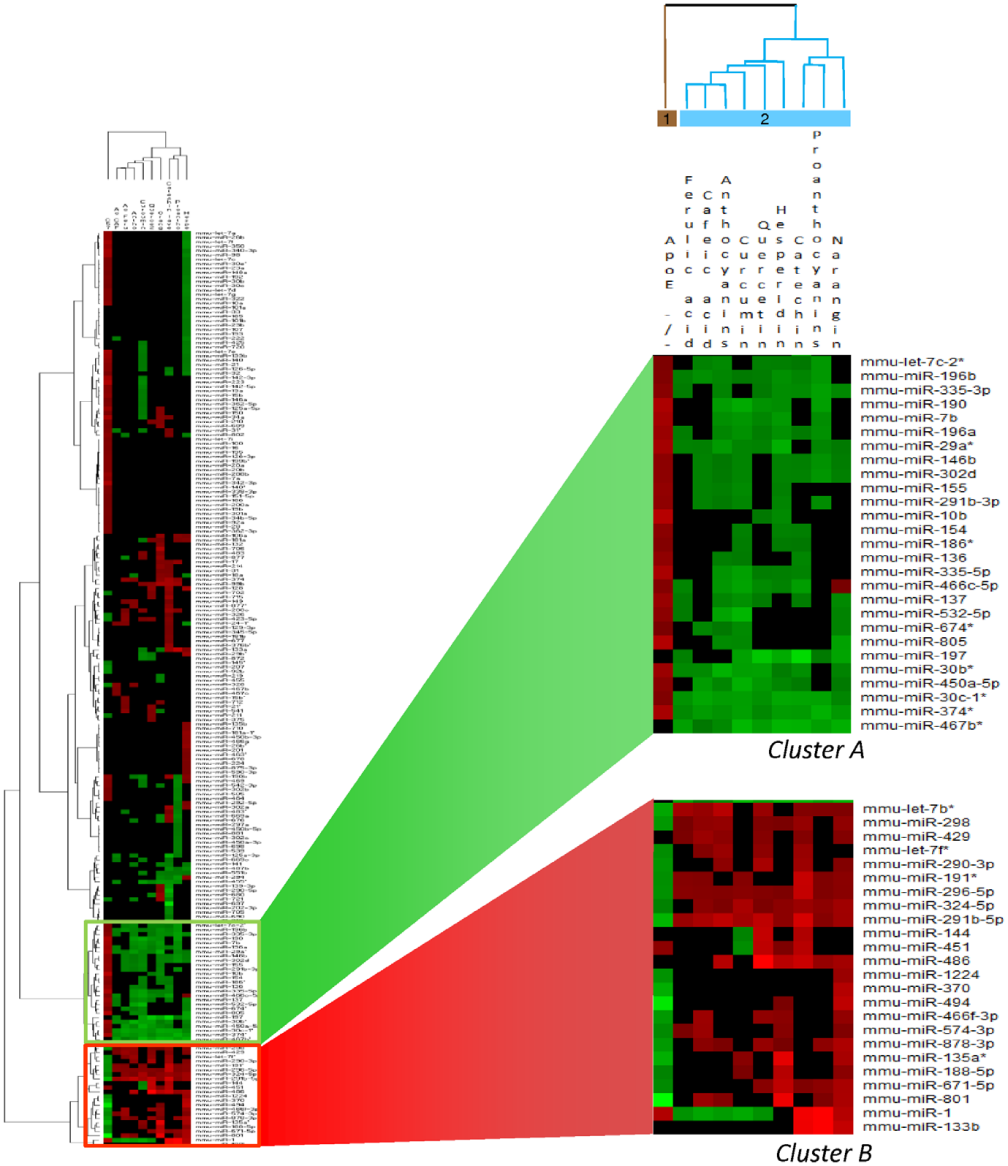
Two-dimensional hierarchical clustering analysis of miRNAs that were significantly altered in at least one of the conditions (apoE−/− compared to wild type, apoE−/− supplemented with polyphenols compared to apoE−/− control diet). miRNA are represented on the vertical axis while different tested conditions are plotted on the horizontal axis. Red or green colors indicate upregulation and downregulation, respectively, while black indicates no changes in the levels of miRNA. Amplified clusters represent miRNA that were either down-regulated (cluster A) or upregulated (cluster B) by the different polyphenols.

A two-dimensional hierarchical clustering analysis was carried out for both miRNAs that were altered in a statistically significant manner as well as the different experimental conditions. Altogether, 10 different conditions were tested in this study: the impact of apolipoprotein E mutagenesis compared to wild-type mice and the effect of nine different polyphenols supplemented in the diet of apoE−/− mice compared to the control diet. Cluster analysis showed two main branches, noted node 1 and node 2 (Figure 2). The miRNA expression profile of the apoE−/− mice, when compared to wild-type, was clustered independently of polyphenol supplemented ones (node 1, Figure 2), suggesting a specific miRNA expression profile induced by apoE mutagenesis. All liver samples from apoE mice that received polyphenols in the diet were grouped together in node 2, an observation that suggest similar miRNA expression profile in livers after polyphenol supplementation in diets.

For the comparison of miRNA expression level (vertical lines), two clusters were identified: noted cluster A and cluster B (Figure 2). These two clusters gather miRNA for which the expression was regulated by most or all of the tested polyphenols. Cluster A corresponds to the miRNA for which the expression was observed to be down-regulated by the polyphenols supplemented in the diet, whereas cluster B encloses miRNA for which the expression was mostly up-regulated. Interestingly, in cluster A, 3 miRNAs (mmu-miR-30c-1*, mmu-miR-374* and mmu-miR-497b*) were identified as being down-regulated by all nine polyphenols tested, while in cluster 2, 2 miRNAs (mmu-miR-291b-5p and mmu-miR-296-5p) were observed as up-regulated by all nine polyphenols (Table 2). Together with the five miRNAs regulated by all polyphenols, clusters also revealed five miRNAs that were modified in their expression by eight polyphenols and nine miRNAs that were modulated by seven different polyphenols. For these 19 miRNAs, regulated by at least seven different polyphenols, twelve were found to be down-regulated and five were up-regulated. For miR-1 and miR-466c-5p, the distribution of the fold change is up- and down-regulated according to the molecule of polyphenol.

**Table 2:** Expression profile of five miRNAs regulated by all nine polyphenols.

	APO E−/−	Quercitin	Hesperidin	Naringin	Anthocyanin	Catechin	Proanthocyanin	Caffeic acid	Ferulic acid	Curcumin
mmu-miR-291b-5p	−3,53	3,9	2,65	2,02	3,95	5,19	1,87	2,43	3,05	2,75
mmu-miR-296-5p	−2,19	1,79	1,95	1,62	1,78	4,22	1,72	1,93	1,84	1,94
mmu-miR-30c-1*	1,72	−2,21	−1,88	−2,92	−2,54	−2,82	−2,77	−2,25	−2,89	−2,59
mmu-miR-467b*	2,66	−2,61	−3,24	−3,46	−1,94	−3,34	−2,63	−2,66	−1,5	−3,57
mmu-miR-374*	2,93	−1,93	−2,39	−2,55	−2,22	−2,47	−3,31	−1,96	−2,52	−2,63

Interestingly, the expression profile of miRNA of apoE−/− mice when compared to wild-type seems to be opposite to that of mice that received polyphenols in their diet. For example, in cluster A, miRNA were identified as up-regulated by mutation in apoE genes when compared to wild type. In apoE−/− mice after polyphenol ingestion for two weeks, the same miRNAs were identified as differentially expressed but their expression was observed to be down-regulated. This inverse expression of miRNA in apoE−/− mice and mice that received polyphenols in their diet was also observed in the cluster 2 (miRNA down-regulated in apoE−/− mice were up-regulated by polyphenol consumption). Among the 27 miRNAs identified as down-regulated in apoE−/− mice when compared to wild-type mice, expression of 24 was up-regulated by polyphenol supplementation; for two miRNAs, the expression was identified as up-regulated by all nine polyphenols, for one by eight polyphenols and two by seven polyphenols. Similarly, for 92 miRNAs up-regulated in apoE−/− mice compared to wild-type ones, the expression was identified as down-regulated for 68 of them after polyphenol supplementation in diet; for two of the miRNAs, the expression was down-regulated by all nine polyphenols, eight of them were down-regulated by eight different polyphenols and five miRNAs were down-regulated by seven polyphenols.

### Identification of miRNA targets and pathways possibly affected by miRNAs

**Figure 3:**
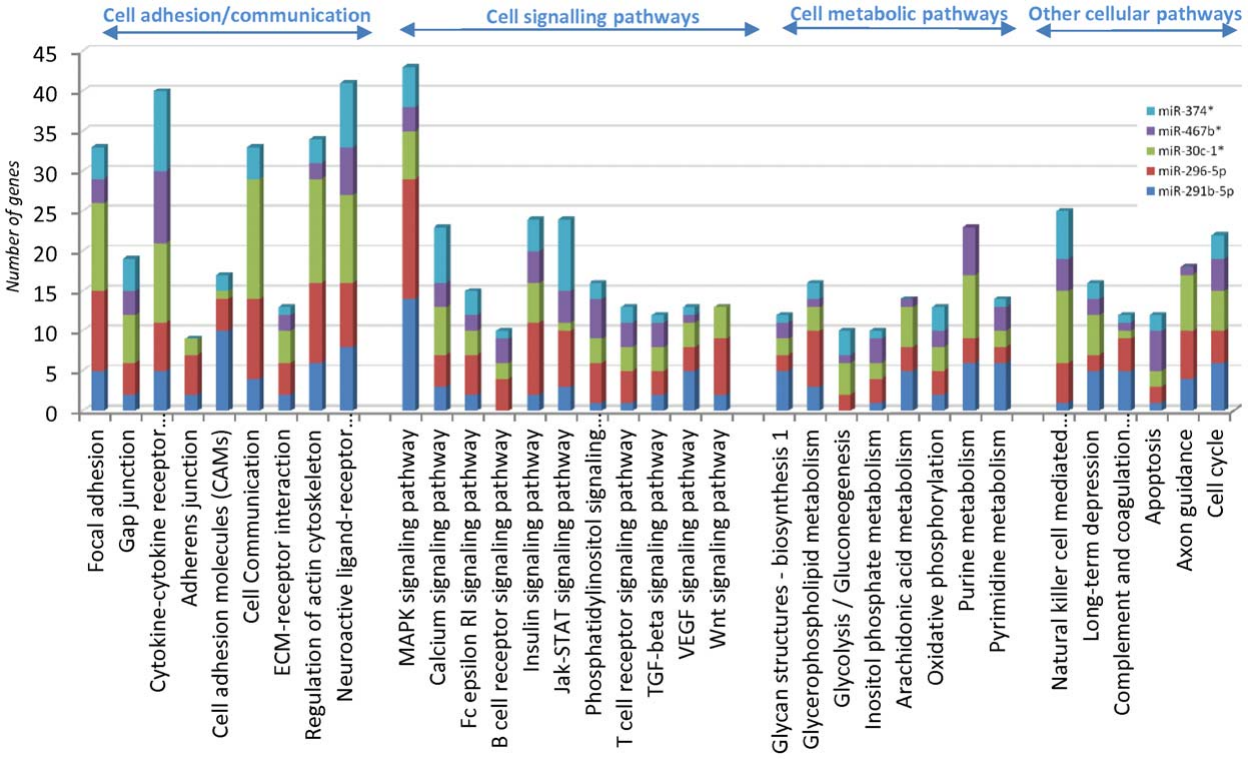
Comparison of pathway signatures obtained from differentially expressed genes. A: hierarchical analysis of the number of genes in each pathway for each condition. Numbers of genes in a pathway are presented on vertical lines while different tested conditions are plotted on the horizontal axes. Yellow color intensity is dependent on the number of genes in each pathway for each condition (the brighter the yellow color the higher the number of genes); B: potential functions of commonly identified pathways and histological presentation of the number of genes in a pathway.

To determine biological meaning of the five miRNAs commonly regulated by all nine polyphenols, potential target genes based on sequence comparison were selected using the MIRANDA database (Sanger institute: http:/microrna.sanger.ac.uk/). Approximately between 500 and 1000 candidate genes were found for each miRNA (complete list and number of target genes can be found in Table S3). We further submitted each list of target genes into the KEGG database to classify the genes into pathways (these pathways are presented in Table S4). Interestingly, among the pathways identified, 34 were common with the potential target genes for all five miRNAs. A large number of them are implicated in cellular processes such as cell adhesion, communication and signaling pathways (Figure 3).

### Microarray analysis of expression of mRNA in liver

To identify the impact of the nine polyphenols as well as the apoE mutation on expression of genes, transcriptome analyses were performed by relative quantification of mRNA isolated from the same tissue sample as for miRNA analysis using pangenomic oligonucleotide microarrays (Table 3). The number of differentially expressed genes varied from 929 to 4101, with highest number observed after hesperidin supplementation while catechin induced the lowest change in the gene expression profile in the liver. Mutation in the apolipoprotein E gene induced gene expression changes in 1388 genes. A list of identified differentially expressed genes is presented in Table S5. On average, the fold-change observed was 1.48 for up-regulated genes and 0.71 for down-regulated genes (Table S6). Differentially expressed genes were placed into cellular pathways using the KEGG database to identify potential biological processes modulated by each polyphenol supplementation in mice diet (Table S7). Two clusters were identified that grouped pathways commonly identified in all conditions (Figure S3). More detailed analysis revealed that there are 54 pathways commonly observed for all nine polyphenols (Table S8). These pathways are mainly involved in cellular processes such as cell adhesion and structure, cellular signaling and metabolic processes to a lesser degree (Figure S4).

**Table 3:** Number of up- and down-regulated mRNAs in the livers of apoE−/− versus C57BL/6 (wild-type mice) and apoE−/− mice after polyphenol supplementation for two weeks.

	Up-regulated	Down-regulated	Total
ApoE−/− *vs* C57BL/6	702	686	1388
Quercetin	755	793	1548
Hesperidin	2076	2025	4101
Narangin	440	513	953
Anthocyanin	1966	1504	3470
Catechin	460	469	929
Proanthocyanin	729	824	1553
Caffeic acid	521	465	986
Ferulic acid	1830	1608	3438
Curcumin	591	565	1156

## Discussion

The major finding of the study is that dietary polyphenols at nutritional doses modify the expression of miRNA *in vivo*. The effects of polyphenol on mRNA expression have already been reported in different *in vitro* or *in vivo* studies, suggesting that some of the biological effects of the compounds could be mediated by their capacity to modulate expression of genes. On the contrary, no studies have been reported on their impact on miRNA expression *in vivo*. In our study, we used a global approach to observe miRNA expression modifications in apoE−/− mice. These results suggest that when supplemented in the diet at a dose that is nutritionally achievable, polyphenols could regulate cellular functions by modulating the expression of miRNA.

Very few studies have investigated the impact of polyphenols on miRNA expression. One of them analyzed the effect of a supra-physiological concentration of epigallocatechin gallate (EGCG) on miRNA expression in human hepatocellular carcinoma HepG2 cells [25]. Using miRNA microarray analysis, the authors observed that EGCG at 100 µM modified the expressions of miRNAs, 13 were identified as up-regulated and 48 were down-regulated. In our study we observed that catechin, aglycon form of EGCG, modulated expression of 44 miRNA among which 14 have been observed as regulated by EGCG in vitro in the above study in the same manner, that is up-regulated or down-regulated in-vitro and in-vivo in our study. Another study revealed, *in vitro*, that curcumin at 10 µM concentration modulated miRNA expression in human hepatocellular carcinoma HepG2 cells [26]. The authors identified 29 differentially expressed miRNAs among which 13 have been also modulated by curcumin in-vivo in our study in the same manner. Furthermore, one recent study reported that stimulation of RAW264.7 cells with LPS resulted in a 12fold increase in miR-155 as compared to unstimulated macrophages and quercetin counteracted LPS induced increase in miR-155 [27].

It has been reported that mutagenesis in the apoE gene modulates expression of genes in the liver [28]. In our study, we demonstrate that apoE mutation also modulates expression of miRNA. Expression of 119 miRNAs was observed to be affected by this mutation, among which 92 were up-regulated and 27 down-regulated. The number of differentially expressed miRNAs in response to polyphenol supplementation in the diet of apoE−/− mice is lower than the number identified in apoE-deficient mice when compared to the wild type. This is not unexpected; a knock-out of a gene is probably more likely to have greater consequences than a supplementation of polyphenols in the diet. The expression profile of miRNA in apoE−/− mice was compared with the expression profile after polyphenol supplementation in diet. As presented in the Figure 2, all liver samples from apoE mice that received polyphenol in the diet were grouped together in node 2. This observation suggests a relatively high similarity in miRNA expression in livers after polyphenol is supplemented in diets. However, the miRNA expression profile of the apoE−/− mice, when compared to wild-type, was clustered independently of polyphenol supplemented ones (node 1, Figure 2) suggesting a specific miRNA expression profile induced by apoE mutagenesis. This analysis also revealed two groups of miRNA with expression that was regulated by most or all of the tested polyphenols. Five miRNAs were observed to be modulated by all nine polyphenols, three miRNAs were identified as being down-regulated and two were up-regulated (Table 2). These commonly regulated miRNA provide a partial mechanism of gene regulation after polyphenol consumption.

One interesting observation was that polyphenols modulated the expression of miRNA in the livers of apoE−/− mice towards the expression profile that has been observed in wild-type ones. miRNAs in cluster A (Figure 2) were identified as having their expression down-regulated by polyphenols. The same miRNA were also identified as differentially expressed after mutagenesis in apoE, however their expression was identified as up-regulated. We could propose that regarding this observation, up-regulation induced by apoE mutagenesis is thwarted by polyphenols to approach the expression in wild-type. For example, mmu-miR-374* is up-regulated after mutagenesis in apoE mice by a factor of 2.96 comparing to the wild-type mice, however when supplemented by polyphenols, this miRNA is down-regulated in apoE by an average factor of −2.44. The same observation of opposite miRNA expression was also observed for the miRNA that are down-regulated in the liver after mutagenesis and for which an increase in expression was observed after polyphenol supplementation (cluster B in Figure 2). Overall, among 27 miRNA identified as down-regulated by apoE knock-out, expression of 24 of them were up-regulated by at least 1 polyphenol and for 92 that were up-regulated polyphenols down-regulated 68 of them. This possible capacity of polyphenols to reverse gene expression in apoE mice has been already observed for caffeic acid phenethyl ester (CAPE) [29]. The authors observed up-regulation of NF-κB-related genes in the aorta in apoE−/− mice compared to wild-type mice (C57/B6), the expression of which was observed to be down-regulated in apoE−/− after 22 weeks of CAPE supplementation, expression that was close to that observed in wild-type mice. This reverse expression of genes to “wild-type” was associated with significant reduction of aortic atherosclerosis. Therefore, the capacity of polyphenols to counteract the expression of miRNA induced by apoE mutagenesis could present another mode of the action of polyphenols at the molecular level underlying the beneficial effects of polyphenols.

Among the differentially expressed miRNAs identified, five of them were observed to be regulated in common by all polyphenols, presenting an interesting common mechanism of the action of polyphenols. However, functions and roles of these five miRNAs are not yet clearly known. Regarding miR-296-5p, its expression has been observed to be down-regulated in endothelial cells exposed to inflammatory stimulus [30]. Different studies have been reported regarding miR-296 for which the expression has been observed to be down-regulated in the mouse brain after prenatal ethanol exposure and has been shown to be associated with mental retardation [31], or parathyroid cancer tissue [32] or even NIH3T3 cells exposed to UV irradiation that induces apoptosis and necrosis. Contrary to these studies, the expression of this miRNA was identified as up-regulated by polyphenols in our study, suggesting that polyphenols might exert a potential beneficial effect regarding these dysfunctions.

Further analyses have been performed with the objective of predicting potential biological functions modulated by the five miRNAs commonly modulated by all polyphenols. It has been suggested that a single miRNA can regulate several target genes, whilst a single gene can also be regulated by several miRNAs; hence changes in miRNA expression can have profound effects on biological systems. We used TargetScanMouse to generate lists of predicted target genes regulated by our miRNAs of interest. Lists of target genes can be found in Table S4. To extract the biological meaning associated with these large lists of genes, we used the Kyoto Encyclopedia of Genes and Genomes (KEGG) to place them into cellular pathways. Different pathways have been identified and are presented in the Table S4. Interestingly, when comparing these pathways obtained from target lists of five miRNAs, 34 were identified in common (Figure 3). These pathways are implicated in different cellular functions, especially cell adhesion/communication, cell signaling and metabolic processes. These pathways present interesting cellular functions as they could be commonly modulated by any of these polyphenols through regulation of the expression of these miRNAs.

Together with exploration of miRNA expression profiles, we also sought to identify the capacity of these polyphenols to modulate the expression of genes by relative quantification of mRNA using oligonucleotide microarrays. Few previous studies have reported that polyphenols *in vivo* can modulate the expression of genes, such as the impact of flavonoids in the rat colon [33], catechin or anthocyanin-rich extract in the mouse aorta [19], [34], quercetin in rat lungs [35], as well as the effect of proanthocyanidins on endothelial cells *in vitro* [36]. In accordance with these studies, using transcriptome approach we identified that the polyphenols modulated expression of genes in liver *in vivo* at nutritional doses. Hierarchical clustering of differentially expressed genes allowed the identification of two groups of polyphenols (Figure S2). Polyphenols present in each group are not necessarily members of the same class of polyphenols. Group 1, for example, groups expression profiles of two phenolic acids (caffeic and ferulic acids) as well as profiles of two flavonoids (hesperidin and naringenin), while node 2 grouped other flavonoid member polyphenols together with curcumin, a specific family of polyphenols. Regarding these data, we could hypothesize that the genomic and consequently probably the biologic effects of these polyphenols are not likely related to their chemical structure. Therefore, it could be appealing to further analyse the impact of polyphenols on gene expression and the cellular effect to sort them not by their chemical structure but by their biological effects. Functional analysis of the obtained lists of differentially expressed genes has been performed in the same manner as for target mRNA of miRNA. The differentially expressed genes were places into cellular pathways using the KEGG database. In all, 200 pathways have been identified. However, among these pathways, 54 pathways were found to be common for nine polyphenols. These pathways are implicated in different metabolic and cellular processes, such as signaling pathways, fatty acid metabolism, oxidative phosphorylation or processes involved in cell adhesion, cellular cytoskeleton or cell junction. Some of these target pathways have been identified in previous studies; it has been reported that quercetin can modulate expression of genes involved in fatty acid catabolism pathways, like beta-oxidation and ketogenesis; proanthocyanidin modulated expression of genes involved in cell migration and proliferation or cell division [34]; while catechin ou anthocyanin induced modulation in expression of genes involved in different pathways of transendothelial migration.

**Figure 4:**
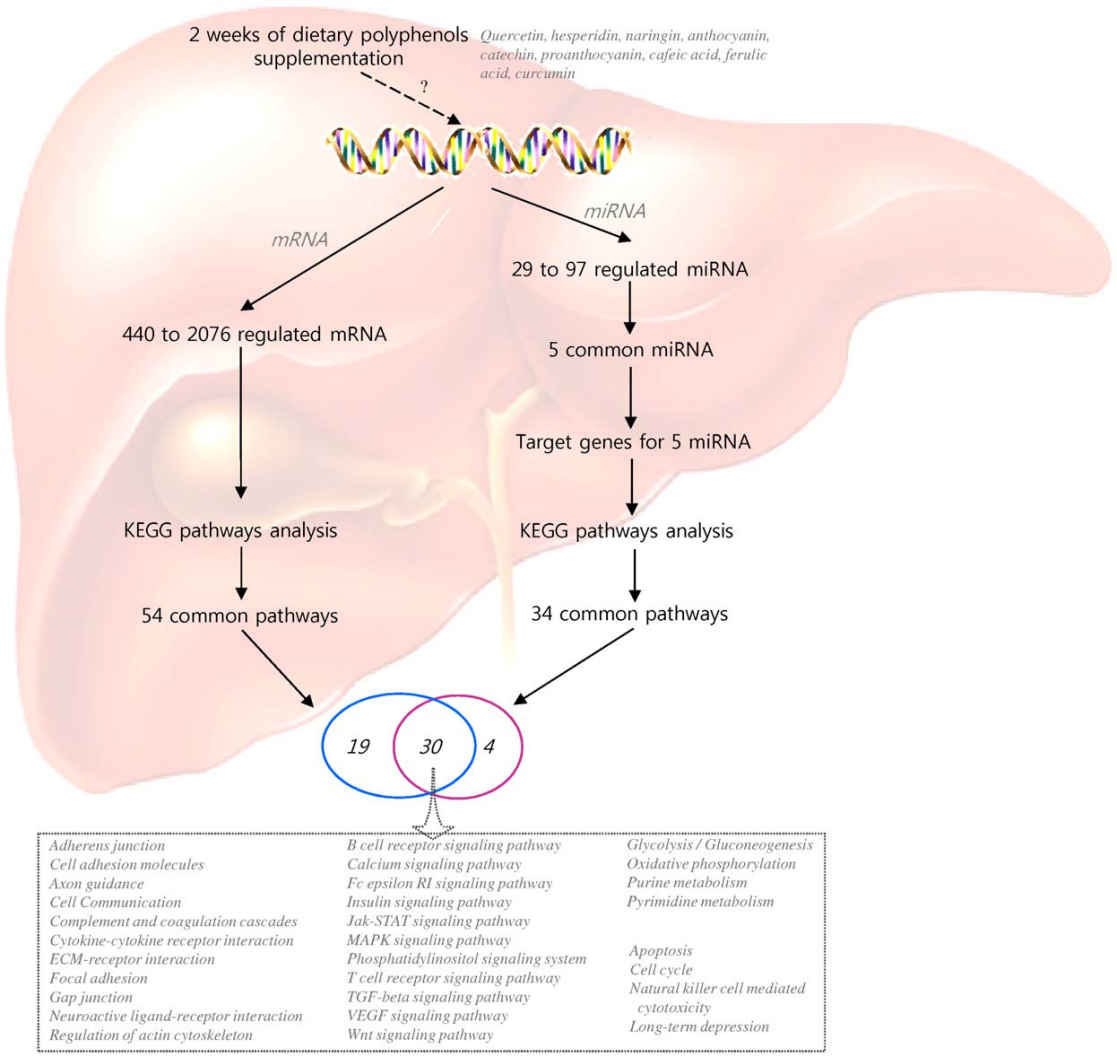
Schematic representation of results of combined miRNA and mRNA microarrays analysis and their potential biological functions.

**Figure 5:**
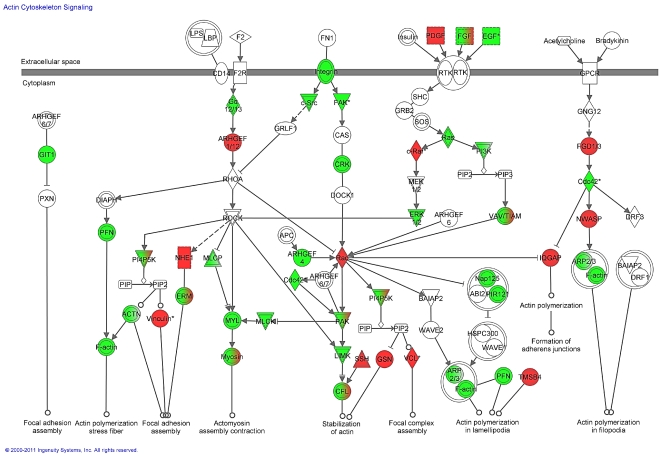
Differentially expressed genes and potential target genes of commonly regulated miRNA involved in actin cytoskeleton signaling pathway obtained using Ingenuity Systems Pathway Analysis. Differentially expressed genes are identified in red; potential target genes of commonly regulated miRNA represented in red and genes that are identified as differentially expressed and which are potential targets of the 5 miRNA are presented by degradation from red to green.

With the objective to identify potential biological targets of polyphenols at both the miRNA and mRNA level identified to be modulated in this study, we compared common pathways of target genes of differentially expressed miRNA with common pathways identified from differentially expressed mRNA. Among 54 and 34 pathways identified respectively from mRNA and miRNA results, 30 were found to overlap (Figure 4). These pathways were found to be involved in different cellular processes such as adherent junctions, cell communication, regulation of the actin cytoskeleton, focal adhesion, signaling pathways such as the MAPK signaling pathway, the calcium signaling pathway, the insulin signaling pathway, as well as different metabolic processes including purine metabolism, pyrimidine metabolism or oxidative phosphorylation. Identification of these common pathways presents a cellular function that could be regulated at both mRNA and miRNA level and presents interesting function targets of polyphenols. Among the 30 pathways identified in common between miRNA target genes and differentially expressed mRNA, some of them are implicated in different steps of leukocyte adhesion and diapedesis, such as actin signaling pathway (Figure 5) as well as focal adhesion, gap junctions or adherent junctions (Figure S5). Hepatic infiltration of white blood cells is an acute response to inflammatory signals [37]. The recruitment of circulating white blood cells is initiated by their adhesion to the endothelial lining of the vessel wall, followed by diapedesis, or transmigration across the endothelial monolayer. The adhesion to endothelial cells is mediates by adhesion molecules synthesized by vascular cells and diapedesis requires endothelial cell deformation and contraction that causes adjacent cells to retract from each other, increasing inter-cellular gaps and facilitating the entry of inflammatory cells, the latest also dependant on endothelial gap and adherence junction proteins. From Figure 5, it could be observed that polyphenols modulated expression of genes implicated in the regulation of cellular actin cytoskeleton as well as genes implicated in signaling pathway that control acto-myosin stress fiber formation. Certain genes in the same pathways are also potential target genes of the 5 commonly regulated miRNA. Furthermore, this analysis also revealed genes that could be target of the 5 miRNA and for which microarray analyses revealed variation in mRNA relative quantities after polyphenol consumption. This observation suggests that polyphenols could regulate adhesion and transendothelial migration of blood cell probably by regulating both expression of genes and miRNA involved in these processes. In agreement with this hypothesis, we have observed, in-vitro, that naringenin and curcumine significantly reduces monocyte adhesion to endothelial cells in vitro (Coban et al., submitted [38]). In-vivo in the same animal model, ApoE knock-out mice, catechin, anthocyanin or curcumin significantly reduced atherosclerosis lesion development via a lower monocyte infiltration. The observed effect is probably regulated via modulation of expression of genes implicated in cellular pathway regulating transendothelial migration in aorta, such as focal adhesion, gap junction or cell adhesion molecules, same pathways as observed for miRNA potential target genes [19], [34]. Also, recent studies have revealed the roles of miRNAs in regulation of oxidative stress and inflammation in vascular tissues, the interaction between inflammatory cells and endothelial cells within this tissue [39]. In conclusion, polyphenols, by regulating both miRNA and mRNA expression could modulate cell activity, presenting molecular mechanisms underlying their biological potency in-vivo.”

It has been suggested that in cell culture systems, the target genes of the enriched miRNAs tend to have lower expression levels most likely due to degradation of mRNA once the miRNA is attached [40]. However, miRNA can, and more frequently, induce translational repression [1]. We could suggest that degradation of mRNAs following attachment of miRNA is one mechanism responsible for changes in gene expression after polyphenol supplementation in our studies. In this hypothesis, a down-regulation of miRNA would lead to an upregulation of gene expression and inversely, an up-regulation of miRNA would lead to a down-regulation of gene expression. In this sense, we examined the relationship between the specific directional change in miRNA and the observed gene expression. This analysis has been done on miRNA commonly regulated by polyphenols and for genes and target-genes in common pathways. It has been observed that, for example, quercetin-modulated expression of 12 genes potentially implicated in MAPK pathways. Among these 12 genes, 5 have been identified as potential targets of common miRNA, and for ferulic acid, 17 genes were observed in common among 41 differentially expressed mRNAs localized in this pathway. Among the 17 genes observed between differentially expressed genes that are also possible targets of five commonly regulated miRNAs, only eight were observed to have a negative correlation in expression, that is, for down-regulated miRNA an up-regulation of expression of mRNA was observed and the inverse. Regarding these observations, we performed qRT-PCR for 8 target genes of commonly regulated miRNA; 4 target genes (ATG5, ITGA6, NCKAP1, SARBS1) of the 2 up-regulated miRNA (mmu-miR-291b-5p, mmu-miR-296-5p) and 4 target genes (AKT1, APC, LMO7, MSN) of the 3 down-regulated miRNA (mmu-miR-30c-1*, mmu-miR-467b* and mmu-miR-374*). This analysis revealed that the expression of only 2 genes (ITGA6 and AKT1) presented negative correlations in expression, *i.e.* the expression of ITGA6, potential target gene of the up-regulated miRNAs, was observed as down-regulated and the expression of AKT1, potential target gene of the down-regulated miRNAs, was identified as up-regulated. Regarding this observation, it could be suggested that miRNA can induce mRNA cleavage but also, and probably more frequently, repress protein synthesis without affected mRNA level. However, this absence of a negative correlation can be due to the fact that alternate action mechanisms for miRNA exist. The action of miRNAs may not to be reflected at the level of their target mRNAs, as they are believed to block or attenuate translation of mRNAs to protein. In such conditions, miRNAs will exert their regulatory role on the level of translation. Moreover, a new mode of the action in which the miRNAs act as positive regulators has also been defined recently; depending upon the state of a cell, an miRNA can act as positive or negative regulator [41]. It is important to remember that regulation of expression of mRNA by polyphenols could also be regulated by modulation of signaling pathway activity by polyphenols. In this way, for example, an inactivation of a transcription factor, such as Nf-κB, could induce a decrease in gene transcription, and consequently mRNA quantity, even though the expression of target miRNA increases. Further studies are required to undergo a more detailed identification of regulatory mechanisms modulated by polyphenols.

In conclusion, we used the microarray approach to evaluate levels of both miRNA and mRNA in the livers of apolipoprotein E-deficient mice after dietary polyphenol supplementation. For the first time, we show that polyphenol supplementation at nutritional doses can modulate the expression of miRNA *in vivo*, and we identified five miRNAs that are commonly modulated by all the polyphenols tested, suggesting that they constitute specific miRNA targets. Interestingly, the expression of these miRNAs seems to be opposite to their expression observed in apoE−/− mice when compared to wild type ones, which provides evidence that polyphenols could counteract the modulation of miRNA-induced apoE knock-out. Different biological pathways, obtained with miRNA target genes, were identified for all of the five miRNAs. Thirty of these pathways were in common with the pathways of modulated mRNA in response to dietary polyphenol supplementation. Taken together, these results suggest interesting cellular targets of polyphenols than could be regulated at both the mRNA and miRNA level.

## Supporting Information

Figure S1 **Chemical structures of polyphenols studied in this work.**
(PDF)

Figure S2 **Hierarchical clustering of mRNA expression profiles.**
(PDF)

Figure S3 **Comparison of pathway signatures obtained from differentially expressed genes: a hierarchical analysis of the number of genes in each pathway for each condition.** Numbers of genes in a pathway are presented on vertical lines while different tested conditions are plotted on the horizontal axes. Yellow color intensity is dependent on the number of genes in each pathway for each condition (the brighter the yellow color the higher the number of genes).
(TIF)

Figure S4 **Potential functions of commonly identified pathways and histological presentation of number of genes in a pathway.**
(TIF)

Figure S5 **Differentially expressed genes and potential target genes of commonly regulated miRNA localized in pathways involved in adhesion and transendothelial migration.** Pathways were identified using Kyoto Encyclopedia of Genes and Genomes database (KEGG); http://www.genome.jp/kegg/) : A) Focal adhesion pathway; B) gap junction pathway; C) adherens junction pathway; D) cell adhesion molecules. Differentially expressed genes are identified in red; potential target genes of commonly regulated miRNA are represented in green and genes that are identified as differentially expressed and could be target of the 5 miRNA are presented in rose.
(PDF)

Table S1 **Composition of the semi-synthetic diet.**
(PDF)

Table S2 **List of identified differentially expressed miRNAs with their fold-change.**
(PDF)

Table S3 **List and number of target genes of five commonly regulated miRNAs.**
(XLS)

Table S4 **KEGG pathways of identified potential target genes of five commonly regulated miRNAs.**
(PDF)

Table S5 **List of differentially expressed mRNAs.**
(XLS)

Table S6 **Average fold-change and minimum and maximum values of fold-change of differentially expressed mRNAs.**
(PDF)

Table S7 **KEGG pathways of differentially expressed mRNAs.**
(PDF)

Table S8 **Common pathways of differentially expressed mRNAs in liver after supplementation with different polyphenols.**
(PDF)

## References

[1] Bartel DP (2004). MicroRNAs: genomics, biogenesis, mechanism, and function.. Cell.

[2] Carthew RW, Sontheimer EJ (2009). Origins and Mechanisms of miRNAs and siRNAs.. Cell.

[3] Li L, Xu J, Yang D, Tan X, Wang H (2009). Computational approaches for microRNA studies: a review.. Mamm Genome.

[4] Esquela-Kerscher A, Slack FJ (2006). Oncomirs - microRNAs with a role in cancer.. Nat Rev Cancer.

[5] Miska EA (2005). How microRNAs control cell division, differentiation and death.. Curr Opin Genet Dev.

[6] Wang Y, Lee CG (2009). MicroRNA and cancer–focus on apoptosis.. J Cell Mol Med.

[7] Dyrskjøt L, Ostenfeld MS, Bramsen JB, Silahtaroglu AN, Lamy P (2009). Genomic profiling of microRNAs in bladder cancer: miR-129 is associated with poor outcome and promotes cell death in vitro.. Cancer Res.

[8] Eisenberg I, Alexander MS, Kunkel LM (2009). miRNAS in normal and diseased skeletal muscle.. J Cell Mol Med.

[9] Matkovich SJ, Van Booven DJ, Youker KA, Torre-Amione G, Diwan A (2009). Reciprocal regulation of myocardial microRNAs and messenger RNA in human cardiomyopathy and reversal of the microRNA signature by biomechanical support.. Circulation.

[10] Vickers KC, Remaley AT (2010). MicroRNAs in atherosclerosis and lipoprotein metabolism.. Curr Opin Endocrinol Diabetes Obes.

[11] Bonauer A, Boon RA, Dimmeler S (2010). Vascular microRNAs.. Curr Drug Targets.

[12] Pérez-Jiménez J, Neveu V, Vos F, Scalbert A (2010). Systematic analysis of the content of 502 polyphenols in 452 foods and beverages: an application of the phenol-explorer database.. J Agric Food Chem.

[13] Hollman PC, Cassidy A, Comte B, Heinonen M, Richelle M (2011). The biological relevance of direct antioxidant effects of polyphenols for cardiovascular health in humans is not established.. J Nutr.

[14] Arts IC, Hollman PC (2005). Polyphenols and disease risk in epidemiologic studies.. Am J Clin Nutr.

[15] Scalbert A, Manach C, Morand C, Rémésy C, Jiménez L (2005). Dietary polyphenols and the prevention of diseases.. Crit Rev Food Sci Nutr.

[16] Schroeter H, Heiss C, Balzer J, Kleinbongard P, Keen CL (2006). (-)-Epicatechin mediates beneficial effects of flavanol-rich cocoa on vascular function in humans.. Proc Natl Acad Sci U S A.

[17] Spencer JP (2009). Flavonoids and brain health: multiple effects underpinned by common mechanisms.. Genes Nutr.

[18] Spencer JP (2010). Beyond antioxidants: the cellular and molecular interactions of flavonoids and how these underpin their actions on the brain.. Proc Nutr Soc.

[19] Auclair S, Milenkovic D, Besson C, Chauvet S, Gueux E (2009). Catechin reduces atherosclerotic lesion development in apo E-deficient mice: a transcriptomic study.. Atherosclerosis.

[20] Camargo A, Ruano J, Fernandez JM, Parnell LD, Jimenez A (2010). Gene expression changes in mononuclear cells in patients with metabolic syndrome after acute intake of phenol-rich virgin olive oil.. BMC Genomics.

[21] Drummond MJ, Glynn EL, Fry CS, Dhanani S, Volpi E (2009). Essential amino acids increase microRNA-499, -208b, and -23a and downregulate myostatin and myocyte enhancer factor 2C mRNA expression in human skeletal muscle.. J Nutr.

[22] Davidson LA, Wang N, Shah MS, Lupton JR, Ivanov I (2009). n−3 Polyunsaturated fatty acids modulate carcinogen-directed non-coding microRNA signatures in rat colon.. Carcinogenesis.

[23] Davis CD, Ross SA (2008). Evidence for dietary regulation of microRNA expression in cancer cells.. Nutr Rev.

[24] Caraux G, Pinloche S (2005). PermutMatrix: a graphical environment to arrange gene expression profiles in optimal linear order.. Bioinformatics.

[25] Tsang WP, Kwok TT (2009). Epigallocatechin gallate up-regulation of miR-16 and induction of apoptosis in human cancer cells.. J Nutr Biochem.

[26] Sun M, Estrov Z, Ji Y, Coombes KR, Harris DH (2008). Curcumin (diferuloylmethane) alters the expression profiles of microRNAs in human pancreatic cancer cells.. Mol Cancer Ther.

[27] Boesch-Saadatmandi C, Pospissil RT, Graeser AC, Canali R, Boomgaarden I (2009). Effect of quercetin on paraoxonase 2 levels in RAW264.7 macrophages and in human monocytes-role of quercetin metabolism.. Int J Mol Sci.

[28] Huang GS, Yang SM, Hong MY, Yang PC, Liu YC (2000). Differential gene expression of livers from ApoE deficient mice.. Life Sci.

[29] Hishikawa K, Nakaki T, Fujita T (2005). Oral flavonoid supplementation attenuates atherosclerosis development in apolipoprotein E-deficient mice.. Arterioscler Thromb Vasc Biol.

[30] Dentelli P, Rosso A, Orso F, Olgasi C, Taverna D (2010). microRNA-222 Controls Neovascularization by Regulating Signal Transducer and Activator of Transcription 5A Expression.. Arterioscler Thromb Vasc Biol.

[31] Wang LL, Zhang Z, Li Q, Yang R, Pei X (2009). Ethanol exposure induces differential microRNA and target gene expression and teratogenic effects which can be suppressed by folic acid supplementation.. Hum Reprod.

[32] Corbetta S, Vaira V, Guarnieri V, Scillitani A, Eller-Vainicher C (2010). Differential expression of microRNAs in human parathyroid carcinomas compared with normal parathyroid tissue.. Endocr Relat Cancer.

[33] Luceri C, Giovannelli L, Pitozzi V, Toti S, Castagnini C (2008). Liver and colon DNA oxidative damage and gene expression profiles of rats fed Arabidopsis thaliana mutant seeds containing contrasted flavonoids.. Food Chem Toxicol.

[34] Mauray A, Felgines C, Morand C, Mazur A, Scalbert A (2010). Bilberry anthocyanin-rich extract alters expression of genes related to atherosclerosis development in aorta of apo E-deficient mice.. Nutr Metab Cardiovasc Dis.

[35] de Boer VC, van Schothorst EM, Dihal AA, van der Woude H, Arts IC (2006). Chronic quercetin exposure affects fatty acid catabolism in rat lung.. Cell Mol Life Sci.

[36] García-Conesa MT, Tribolo S, Guyot S, Tomás-Barberán FA, Kroon PA (2009). Oligomeric procyanidins inhibit cell migration and modulate the expression of migration and proliferation associated genes in human umbilical vascular endothelial cells.. Mol Nutr Food Res.

[37] Ramaiah SK, Jaeschke H (2007). Role of neutrophils in the pathogenesis of acute inflammatory liver injury.. Toxicol Pathol.

[38] Chanet A, Milenkovic D, Deval C, Potier M, Constans J (2011). Naringin, the major grapefruit flavonoid, specifically affects atherosclerosis development in diet-induced hypercholesterolemia in mice.. Journal of Nutritional Biochemistry.

[39] Hulsmans M, De Keyzer D, Holvoet P (2011). MicroRNAs regulating oxidative stress and inflammation in relation to obesity and atherosclerosis.. FASEB J.

[40] Sood P, Krek A, Zavolan M, Macino G, Rajewsky N (2006). Cell-type-specific signatures of microRNAs on target mRNA expression.. Proc Natl Acad Sci U S A.

[41] Vasudevan S, Tong Y, Steitz JA (2007). Switching from repression to activation: microRNAs can up-regulate translation.. Science.

