# Supplementary material for: Modulation of miRNA Expression by Dietary Polyphenols in apoE Deficient Mice: A New Mechanism of the Action of Polyphenols
Source: PLoS One. 2012 Jan 10;7(1):e29837. doi: 10.1371/journal.pone.0029837 (PMC3254631; doi:10.1371/journal.pone.0029837)
Supplement: Figure S1 — Chemical structures of polyphenols studied in this work. (PDF) [file pone.0029837.s001.pdf]

Supplement figure S1 : Chemical structures of polyphenols studied in this work.

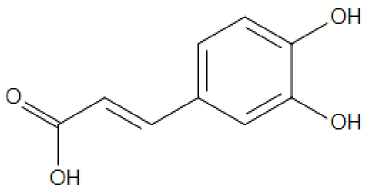

Caffeic acid

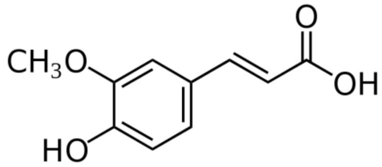

Ferulic acid

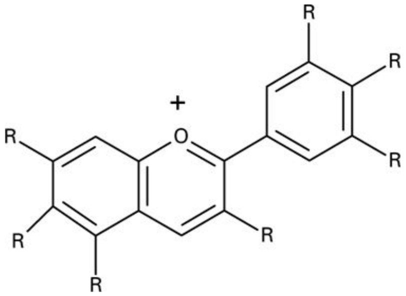

Anthocyanin

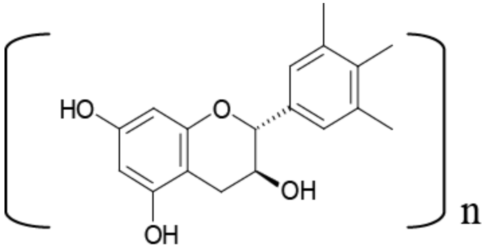

Proanthocyanin

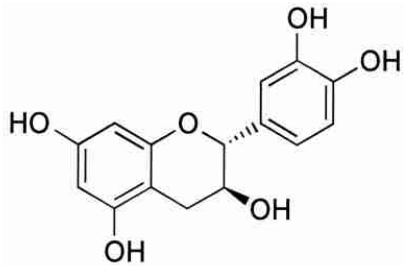

Catechin

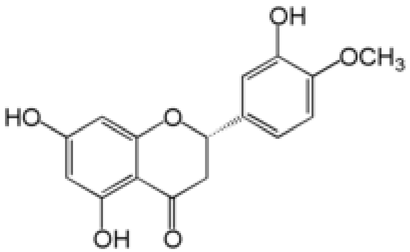

Hesperidin

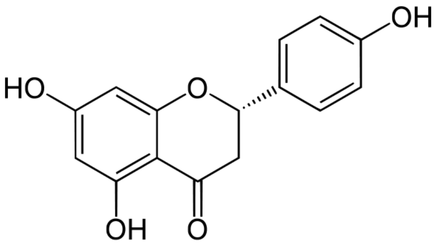

Naringenin

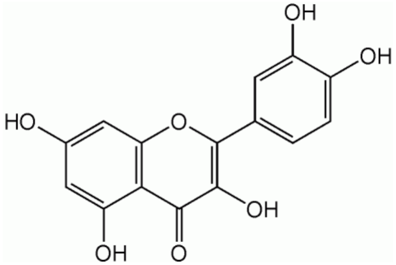

Quercetin

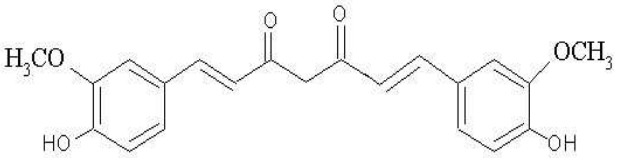

Curcumin
