# Supplementary figures and images for: Modulation of miRNA Expression by Dietary Polyphenols in apoE Deficient Mice: A New Mechanism of the Action of Polyphenols
Source: PLoS One. 2012 Jan 10;7(1):e29837. doi: 10.1371/journal.pone.0029837 (PMC3254631; doi:10.1371/journal.pone.0029837)

**Supplement figure S2 : Hierarchical clustering of mRNA expression profiles**

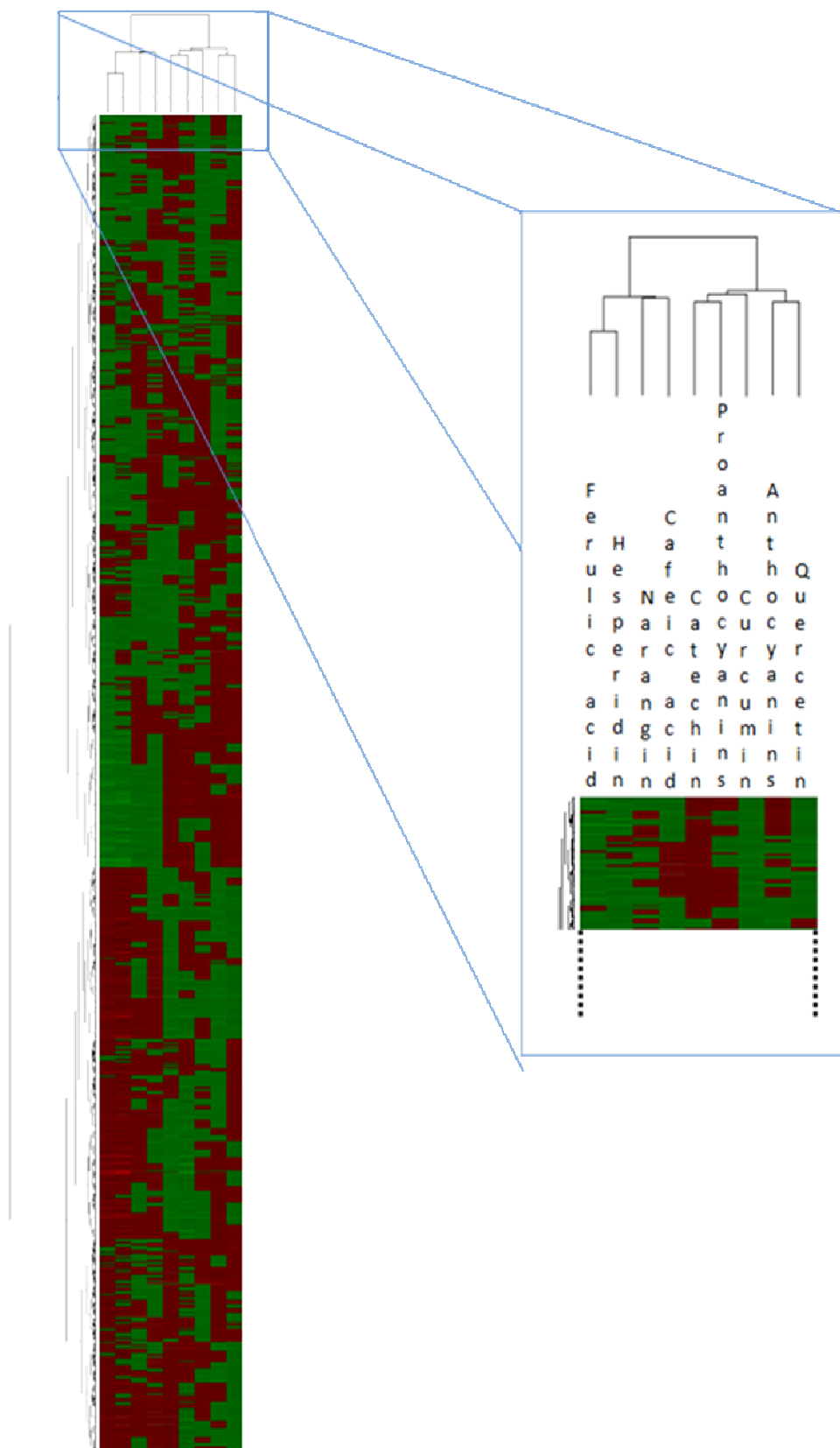

Supplement: Figure S2 — Hierarchical clustering of mRNA expression profiles. (PDF) [file pone.0029837.s002.pdf]

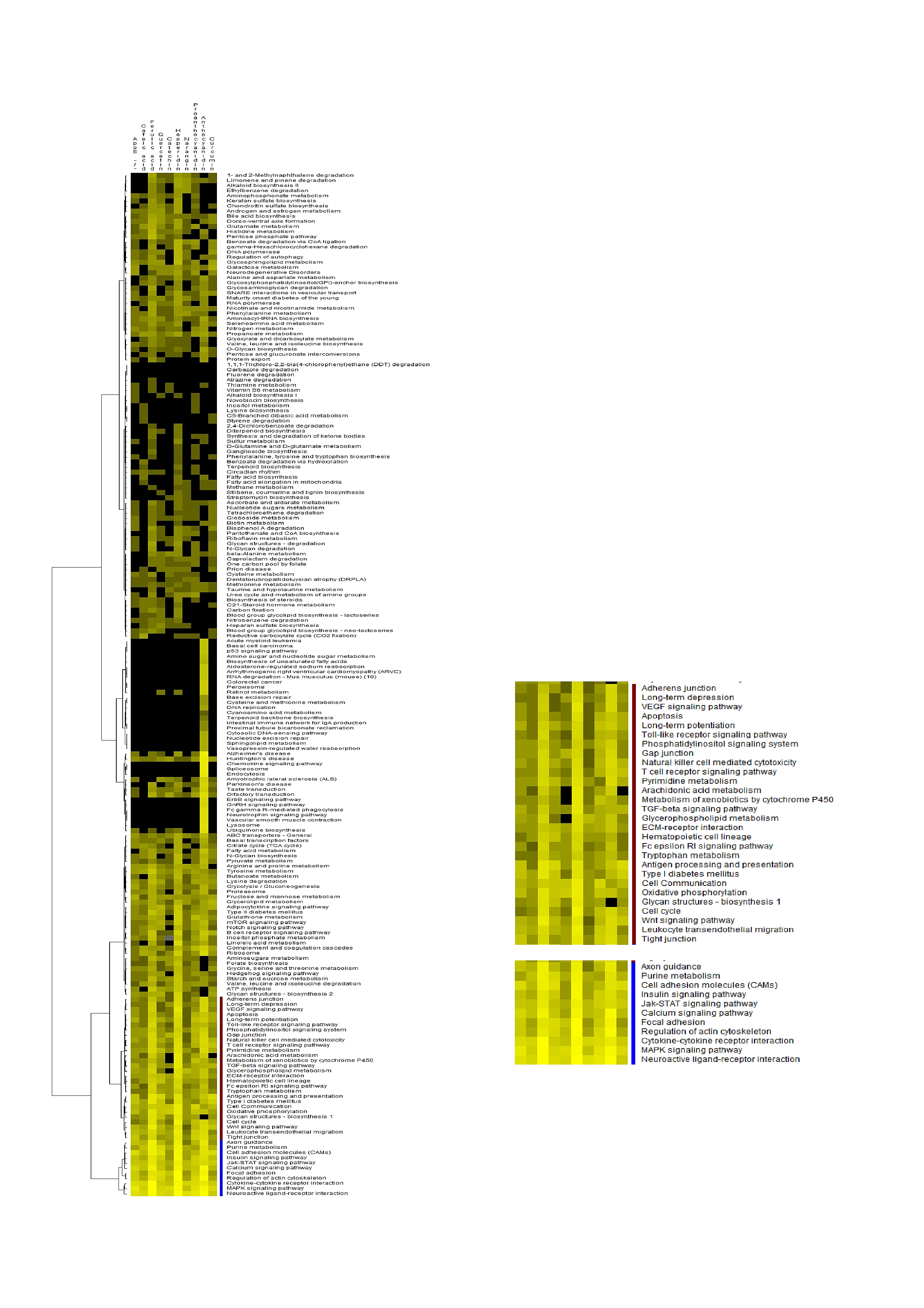

Supplement: Figure S3 — Comparison of pathway signatures obtained from differentially expressed genes: a hierarchical analysis of the number of genes in each pathway for each condition. Numbers of genes in a pathway are presented on vertical lines while different tested conditions are plotted on the horizontal axes. Yellow color intensity is dependent on the number of genes in each pathway for each condition (the brighter the yellow color the higher the number of genes). (TIF) [file pone.0029837.s003.tif]

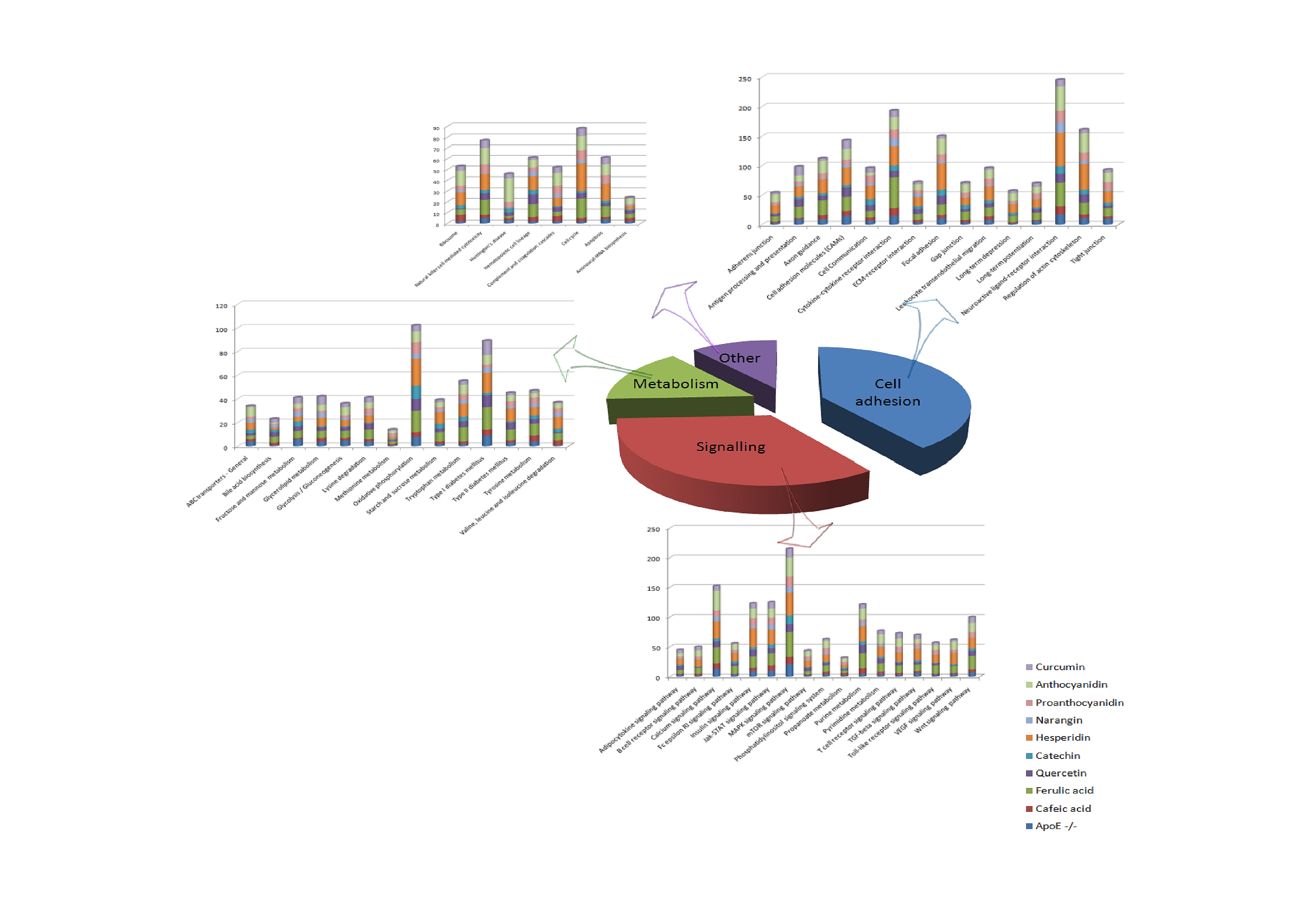

Supplement: Figure S4 — Potential functions of commonly identified pathways and histological presentation of number of genes in a pathway. (TIF) [file pone.0029837.s004.tif]
