# Supplementary material for: Modulation of miRNA Expression by Dietary Polyphenols in apoE Deficient Mice: A New Mechanism of the Action of Polyphenols
Source: PLoS One. 2012 Jan 10;7(1):e29837. doi: 10.1371/journal.pone.0029837 (PMC3254631; doi:10.1371/journal.pone.0029837)

Supplement Figure S5

A) Focal adhesion

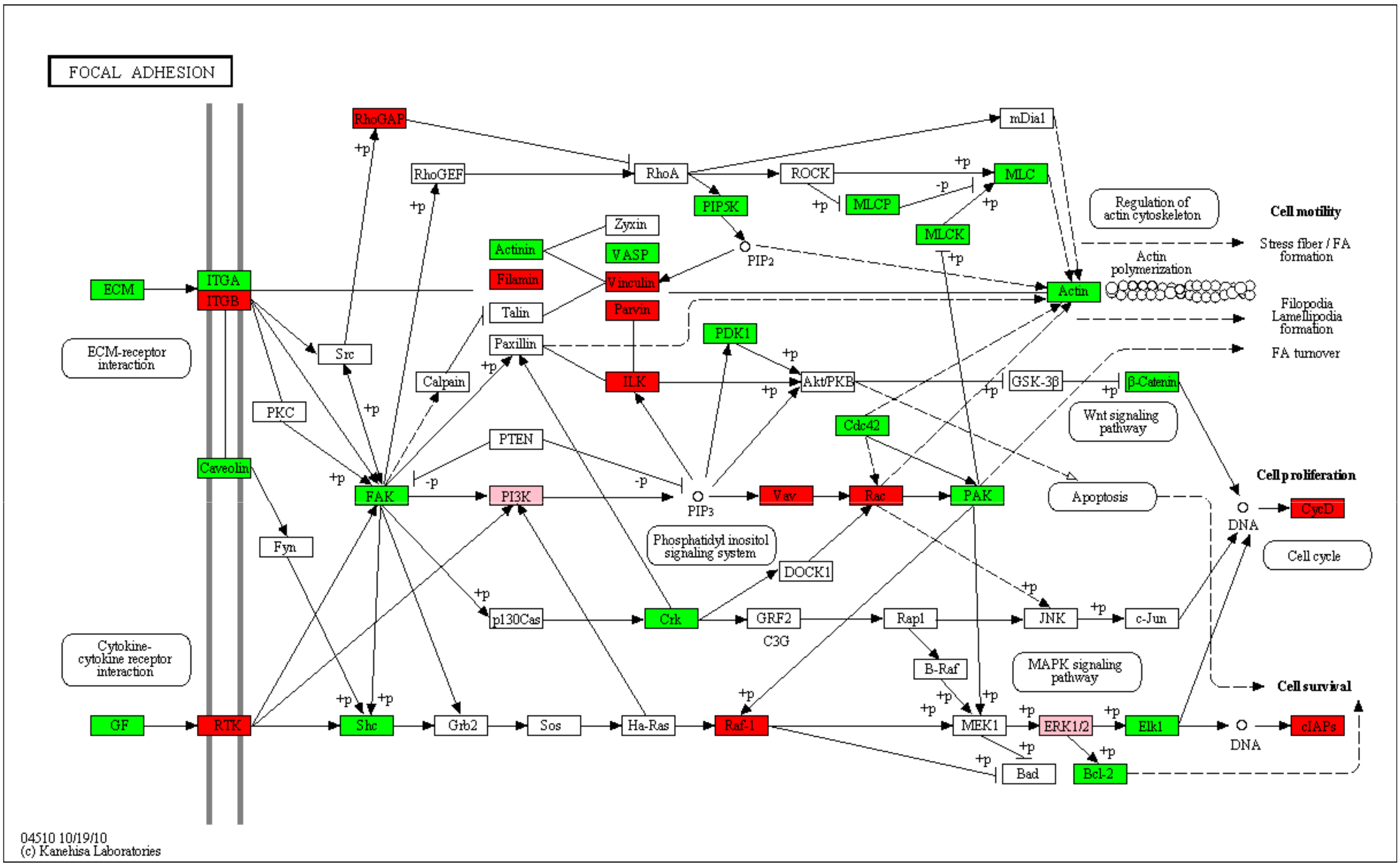

B) Adherens junction pathway

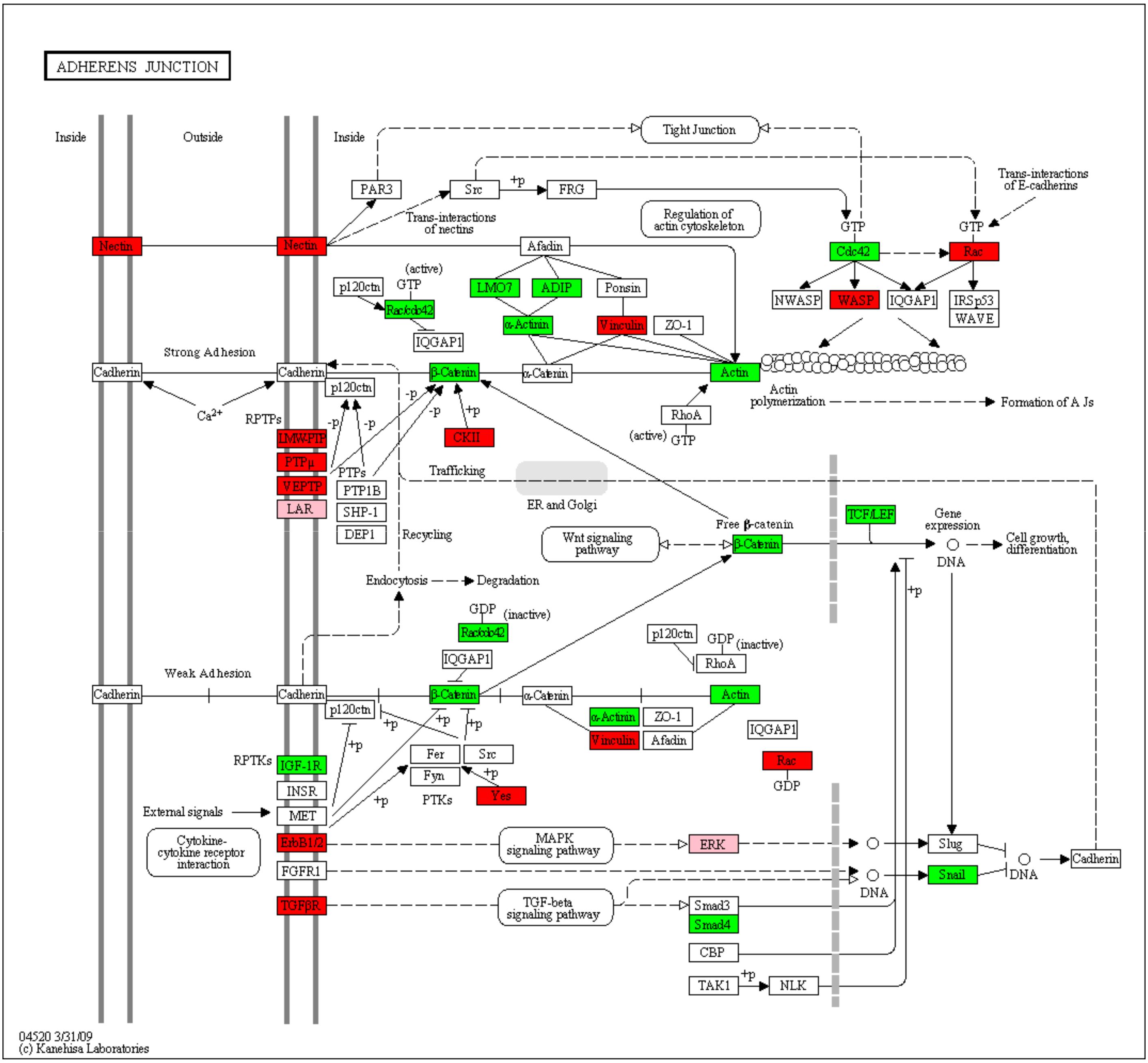

C) Gap junction pathway

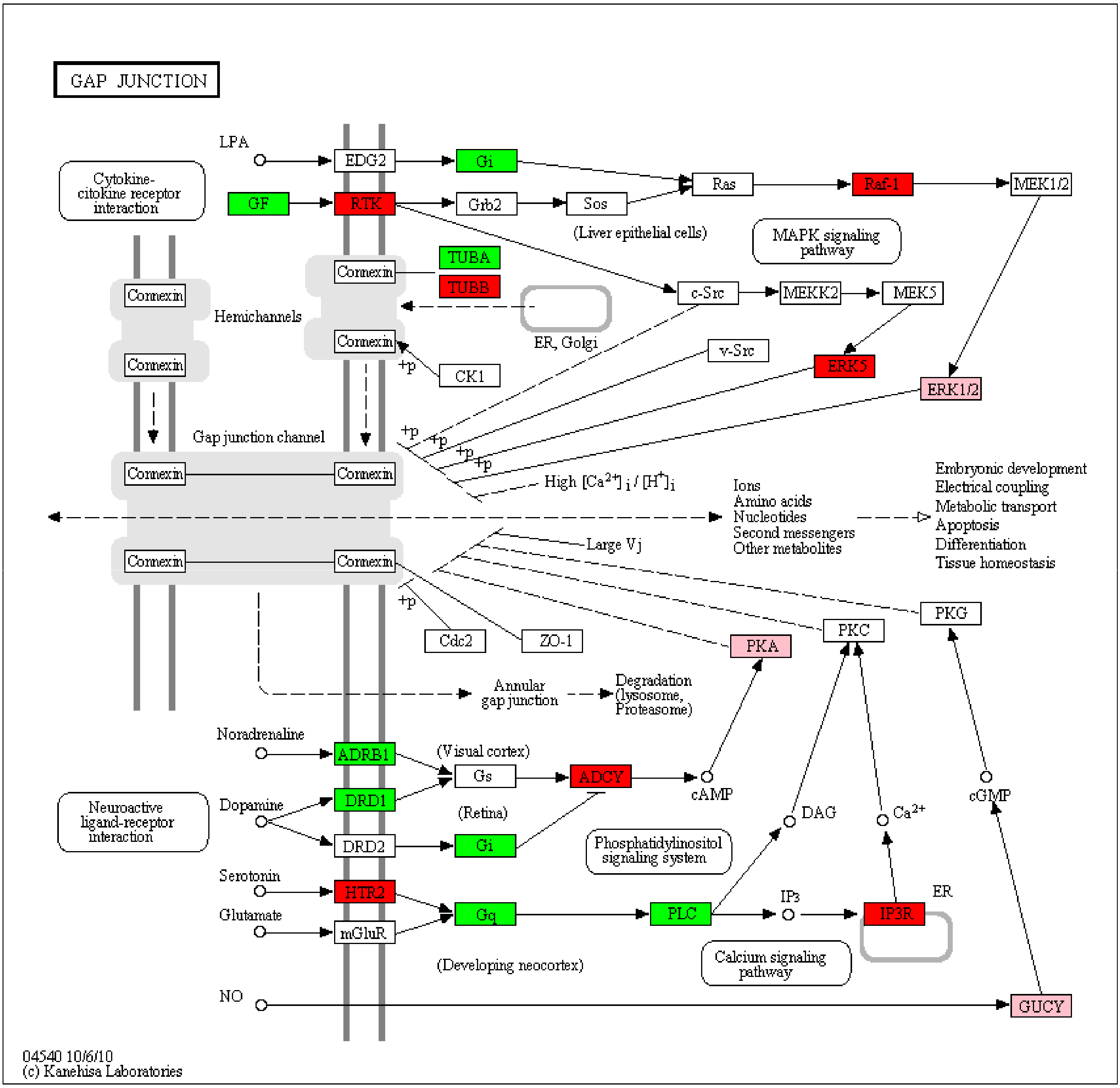

D) Cell adhesion molecules

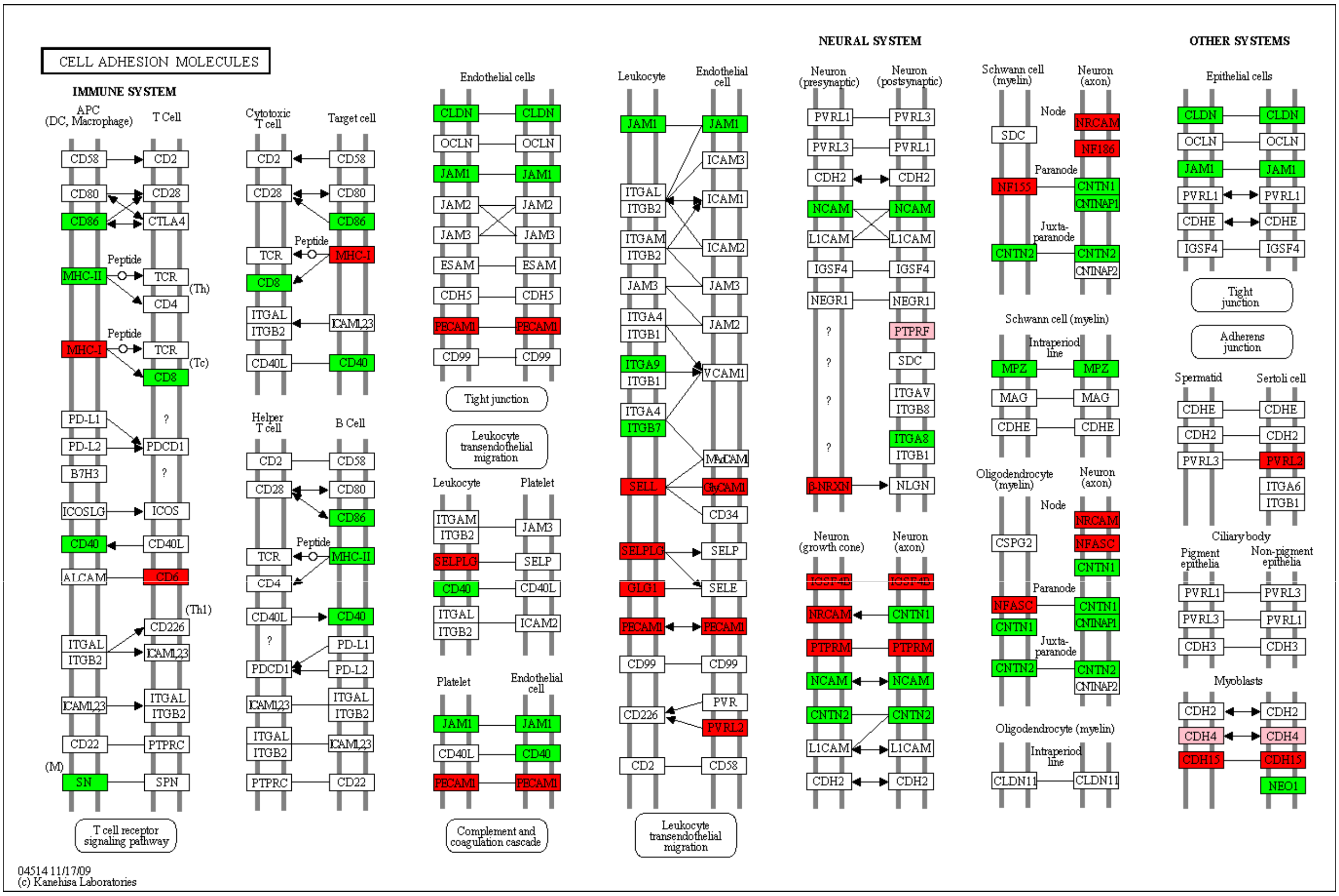

Supplement: Figure S5 — Differentially expressed genes and potential target genes of commonly regulated miRNA localized in pathways involved in adhesion and transendothelial migration. Pathways were identified using Kyoto Encyclopedia of Genes and Genomes database (KEGG); http://www.genome.jp/kegg/) : A) Focal adhesion pathway; B) gap junction pathway; C) adherens junction pathway; D) cell adhesion molecules. Differentially expressed genes are identified in red; potential target genes of commonly regulated miRNA are represented in green and genes that are identified as differentially expressed and could be target of the 5 miRNA are presented in rose. (PDF) [file pone.0029837.s005.pdf]
