# Supplementary material for: Modulation of miRNA Expression by Dietary Polyphenols in apoE Deficient Mice: A New Mechanism of the Action of Polyphenols
Source: PLoS One. 2012 Jan 10;7(1):e29837. doi: 10.1371/journal.pone.0029837 (PMC3254631; doi:10.1371/journal.pone.0029837)
Supplement: Table S1 — Composition of the semi-synthetic diet. (PDF) [file pone.0029837.s006.pdf]

Supplement table S1 : Composition of the semi-synthetic diet

Control diet

Ingredients	Percentage
Wheat Starch	62,95
Casein	20
Corn oil	7
Cellulose	5
Mineral mix AIN-93G	3,5
Vitamin mix AIN-93G	1
L-cystin	0,3
Choline bitartrate	0,25
Total	*100*

Polyphenols **diet**

Ingredients	Percentage
Wheat Starch	62,95
Casein	20
Corn oil	7
Cellulose	5
Mineral mix AIN-93G	3,5
Vitamin mix AIN-93G	1
L-cystin	0,3
Choline bitartrate	0,25
Polyphenol*	
Total	*100*

* - proanthocyanidins, cafein acid or ferulic acid : 0,2%
- curcuin, catechin, anthocyanins, quesrcetin, narengin or hesperidin : 0,02%

Corresponding quantities of polyphenols were deduced from wheat starch amount
