# Supplementary material for: Modulation of miRNA Expression by Dietary Polyphenols in apoE Deficient Mice: A New Mechanism of the Action of Polyphenols
Source: PLoS One. 2012 Jan 10;7(1):e29837. doi: 10.1371/journal.pone.0029837 (PMC3254631; doi:10.1371/journal.pone.0029837)
Supplement: Table S2 — List of identified differentially expressed miRNAs with their fold-change. (PDF) [file pone.0029837.s007.pdf]

Supplement table S2 : List of identified differentially expressed miRNA with their fold-change

ID	ApoE-/-	Cafeic acid	Ferulic acid	Anthocyanin	Catechin	Curcumin	Hesperidin	Narangin	Proanthocyanin	Quercetin
mmu-let-7a	2,15						-1,93			
mmu-let-7b*	-2,92	2,77	2,36	3,63	1,76		1,68		1,72	2,42
mmu-let-7c	1,62						-1,57			
mmu-let-7c-2*	1,65		-1,77	-1,95	-1,53			-1,67	-2,38	-1,70
mmu-let-7d	1,80						-1,87			
mmu-let-7e	2,71					-1,66	-1,67			
mmu-let-7f	2,74						-2,18			
mmu-let-7f*	-2,92		1,80	2,43	2,41					2,87
mmu-let-7g	1,77						-1,78			
mmu-let-7i	1,68									
mmu-miR-1	3,33	-2,23	-2,86	-2,53	7,55	-2,99	3,44		1,13	-2,19
mmu-miR-100	1,77									
mmu-miR-101a							-1,58			
mmu-miR-101b							-1,53			
mmu-miR-106a							1,53	2,25	1,77	
mmu-miR-107							-1,52			
mmu-miR-10a	2,00						-1,60			
mmu-miR-10b	3,55							-1,99		-1,53
mmu-miR-1224	-2,24						2,46			
mmu-miR-125a-3p		-1,83		-1,57	-3,31				-1,56	
mmu-miR-125a-5p	2,53					-1,58		1,76		
mmu-miR-126-3p	1,83									
mmu-miR-126-5p	1,93				-1,56	-1,53	-1,59			
mmu-miR-128					2,35		2,90			2,83
mmu-miR-129-3p					1,53					
mmu-miR-132								1,85		

mmu-miR-133a					2,53	-1,63	1,81	-1,58	4,52	
mmu-miR-133b					7,22		4,13		9,47	
mmu-miR-135a*	-3,24					1,91	2,93	6,31		
mmu-miR-135b							1,85			
mmu-miR-136	2,15			-1,67	-1,86	-1,57				
mmu-miR-137	2,40	-1,68		-1,82	-1,62	-2,16	-1,96			-1,58
mmu-miR-139-3p					-2,49			1,85		
mmu-miR-140	2,17					-1,52	-1,52			
mmu-miR-140*	1,62									
mmu-miR-141				-1,51	-1,68		-1,57	-1,74		
mmu-miR-142-3p	2,75					-1,79				
mmu-miR-142-5p	1,96					-1,74				
mmu-miR-144					3,24	-1,84				4,16
mmu-miR-145*	-1,70									
mmu-miR-146a	1,64					-1,55				
mmu-miR-146b	2,20		-1,74	-1,53	-1,75	-1,82	-1,66	-1,75	-2,22	
mmu-miR-148a	1,53						-1,53			
mmu-miR-149			1,58	1,85	1,54					
mmu-miR-150	1,73					-1,53		1,75		
mmu-miR-151-5p	1,52									
mmu-miR-154	2,00				-1,68	-1,72		-1,74		
mmu-miR-155	2,23		-1,79	-1,69		-2,60		-1,72		
mmu-miR-15b	2,13					-1,58				
mmu-miR-15b*			1,72							
mmu-miR-16	1,72									
mmu-miR-17					1,67			1,75	2,27	
mmu-miR-181a						-1,51	1,77	1,53	1,62	1,60
mmu-miR-181a-1*							2,15			
mmu-miR-181b					1,68					
mmu-miR-185							-1,62			

mmu-miR-186	1,53									
mmu-miR-186*	1,87				-1,52	-1,63		-1,58		
mmu-miR-188-5p	-1,89					1,69	2,00	3,52		
mmu-miR-18a				-1,65	1,75		-1,78	1,89		
mmu-miR-190	3,13			-2,56		-3,34		-2,84	-2,63	-2,22
mmu-miR-190b	2,32			-1,54	2,44	-1,83	1,82		-1,80	
mmu-miR-191*	-2,13	1,85	1,74		4,31	1,82	1,99		1,73	
mmu-miR-192	1,65						-1,69			
mmu-miR-193							-1,53			
mmu-miR-195	1,73									
mmu-miR-196a	2,47	-1,66		-1,87	-1,53	-2,21		-2,15	-2,18	-1,63
mmu-miR-196b	1,73	-1,66	-2,13	-1,87	-1,81	-1,53		-2,16	-2,39	-1,63
mmu-miR-197		-1,72			-5,55	-2,73	-2,82	-3,89	-1,56	-4,36
mmu-miR-199b	2,46					-1,51	-1,56			
mmu-miR-199b*	1,86									
mmu-miR-19a	1,94					-1,57				
mmu-miR-19b	1,54									
mmu-miR-200a	1,54									
mmu-miR-200b	2,35									
mmu-miR-200c			1,52		2,47	1,85			1,79	
mmu-miR-201							1,73			
mmu-miR-202-3p					-1,96					
mmu-miR-207	-1,95									
mmu-miR-20a	1,86									
mmu-miR-20b	1,87									
mmu-miR-21	1,97					-1,52	-1,54			
mmu-miR-21*			1,56							1,83
mmu-miR-210	1,72							1,81		1,66
mmu-miR-211	-1,52									1,66
mmu-miR-212					-2,84					

mmu-miR-214					1,70			2,17		
mmu-miR-219								-1,69		
mmu-miR-222		-1,58					-1,92			
mmu-miR-223	3,26					-1,96				
mmu-miR-224							1,56			
mmu-miR-23a	1,68						-1,54			
mmu-miR-23b							-1,53			
mmu-miR-24-1*		1,66	1,74		1,65		-2,13			
mmu-miR-26b	2,16						-1,89			
mmu-miR-26b*							2,22			
mmu-miR-28	1,57									
mmu-miR-290-3p	-2,27			2,62	2,56		1,73			2,99
mmu-miR-290-5p					-3,48			1,67		
mmu-miR-291b-3p	2,13		-1,82	-1,59		-1,89		-1,83	-2,27	
mmu-miR-291b-5p	-3,53	2,43	3,46	3,95	5,19	2,75	2,65	2,19	1,87	3,93
mmu-miR-292-5p							1,57		-1,57	
mmu-miR-294					-1,66		2,94	-1,92	-2,49	
mmu-miR-296-5p	-2,19	1,93	1,84	1,78	4,22	1,95	1,95	1,62	1,72	1,79
mmu-miR-297a									-1,55	
mmu-miR-298	-2,39	1,52	2,17	2,27	2,31		2,15	1,55		1,69
mmu-miR-29a*	2,82	-1,75	-1,73	-2,49		-1,83	-1,56	-3,25	-1,89	-1,75
mmu-miR-29b*						-2,40	2,44			-1,65
mmu-miR-301a	1,54									
mmu-miR-302a					2,53		1,80		-1,51	
mmu-miR-302b	1,56								-1,76	
mmu-miR-302c									-1,63	
mmu-miR-302d	2,73		-1,79	-1,57	-1,75	-1,87	-1,69	-1,87	-2,35	
mmu-miR-30b	1,59						-1,69			
mmu-miR-30b*	2,57	-1,94	-1,99	-3,37	-2,97	-2,64	-1,80	-1,65		-2,72
mmu-miR-30c	1,53						-1,81			

mmu-miR-30c-1*	1,72	-2,25	-2,89	-2,54	-2,82	-2,59	-1,88	-2,92	-2,77	-2,29
mmu-miR-30e*	1,63						-1,58			
mmu-miR-31					1,63			1,63		
mmu-miR-31*	1,72	-1,53			1,54	-1,55				
mmu-miR-32	1,58				-1,77		-1,80			
mmu-miR-322	1,80						-1,67			
mmu-miR-324-5p	-1,73		2,11	1,65	2,52	2,99	2,68	2,22	2,88	1,65
mmu-miR-326	-1,67		1,86		1,52					
mmu-miR-328	-1,94	1,79		1,69						
mmu-miR-33							-1,57			
mmu-miR-335-3p	1,94	-1,58		-1,79	-1,99		-1,93	-2,58	-2,67	-1,55
mmu-miR-335-5p	2,99			-2,27	-1,78	-2,69		-1,66		-1,63
mmu-miR-338-3p	1,62									
mmu-miR-340-3p	2,86						-1,63			
mmu-miR-342-3p	3,20									
mmu-miR-345-5p					1,59					
mmu-miR-34a	2,94				1,55	-1,68		1,86		
mmu-miR-34b-5p	1,52									
mmu-miR-350	2,35						-2,17			
mmu-miR-362-3p	1,56									
mmu-miR-362-5p	1,73					-1,52				
mmu-miR-370	-3,22						3,43			
mmu-miR-374			2,33	1,90	2,90			2,48	2,13	
mmu-miR-374*	2,93	-1,96	-2,52	-2,22	-2,47	-2,63	-2,39	-2,55	-3,31	-1,93
mmu-miR-375										1,58
mmu-miR-376b*					2,27					
mmu-miR-423-5p	-1,69		1,96		1,79	1,54				1,70
mmu-miR-425						-1,57	-1,62			
mmu-miR-429		1,63	2,56	2,48	1,78		1,81			1,84
mmu-miR-450a-3p									-1,67	

mmu-miR-450a-5p	1,96		-2,51	-1,86	-1,58	-3,30	-1,56	-1,86		-2,41
mmu-miR-450b-3p							2,90			
mmu-miR-450b-5p									-1,51	
mmu-miR-451	2,53				3,68	-2,57		1,64		3,87
mmu-miR-455								-1,67		
mmu-miR-455*		-1,85			-2,33	-2,49		-2,48	-2,37	-1,82
mmu-miR-463*							1,70			
mmu-miR-464	1,64								-1,85	
mmu-miR-466c-5p	1,80	-1,85		-2,35	-2,67	-2,79	1,53	-1,77		-1,98
mmu-miR-466f-3p	-2,46		2,56				2,25	1,99		2,16
mmu-miR-466g							2,20			
mmu-miR-467b		1,62								
mmu-miR-467b*		-2,66	-1,53	-1,94	-3,34	-3,57	-3,24	-3,46	-2,63	-2,61
mmu-miR-467c		1,72								
mmu-miR-469	1,74					-1,56	1,64		-1,97	
mmu-miR-483						1,65		1,89		
mmu-miR-483*			-1,56		1,56				-1,73	
mmu-miR-486				3,47	4,45		2,82	3,65	2,34	1,28
mmu-miR-487b					-2,16		-2,92		-1,51	
mmu-miR-494	-6,46						3,94	1,54		
mmu-miR-505	1,57								-1,78	
mmu-miR-532-5p	2,40	-1,59		-2,81		-3,53	-1,78			
mmu-miR-539									-1,65	
mmu-miR-541			1,90					-1,59		1,62
mmu-miR-542-3p	1,74					-1,57		-1,52	-2,00	
mmu-miR-551b					-1,56	-1,67	-1,52	-1,62	-1,56	
mmu-miR-574-3p	-2,39	1,61	2,49				1,72	1,87		
mmu-miR-590-3p							1,54			
mmu-miR-669a					1,95			-1,89	-2,27	
mmu-miR-669c		-1,54			-1,53	-1,54				

mmu-miR-671-5p	-2,13				1,70		2,99	3,90	2,39	1,94
mmu-miR-674*	1,53		-1,89	-1,53		-1,98	-1,80			
mmu-miR-676				-1,52	1,87			-1,75	-1,74	
mmu-miR-677					1,64					
mmu-miR-678							1,63			
mmu-miR-680					-2,60			2,31		-1,82
mmu-miR-689	1,54							1,99		
mmu-miR-690					-1,55					
mmu-miR-697					-5,89					
mmu-miR-698									-1,67	
mmu-miR-702	-1,58				1,95					1,66
mmu-miR-705					-1,91					
mmu-miR-706								3,27		
mmu-miR-710							1,83			
mmu-miR-712			1,63					1,57		
mmu-miR-715					2,53					1,70
mmu-miR-720						-1,68	-1,67			
mmu-miR-721	-1,59			-1,76	-1,75			2,46		
mmu-miR-7a	2,56									
mmu-miR-7b	2,82			-2,14		-2,34		-2,46	-2,65	-1,86
mmu-miR-801	-9,72		2,45	1,74			3,42	7,20	1,68	
mmu-miR-802	1,99		-1,55		1,70		-1,69			
mmu-miR-805	3,37			-1,98		-1,92	-2,76			
mmu-miR-872						-1,96				-1,70
mmu-miR-875-3p							1,55			
mmu-miR-877			-1,87			1,55		2,35		
mmu-miR-877*				1,89	1,96					
mmu-miR-878-3p	-2,43		2,88	1,61			3,18	1,53	2,56	2,33
mmu-miR-881									-1,54	
mmu-miR-92a	1,60									

mmu-miR-92b	-1,95									
mmu-miR-98	2,69						-1,86			
mmu-miR-99b					2,94	1,76		3,00	2,56	1,95

max FC up	3,55	2,77	3,46	3,95	7,55	2,99	4,13	7,20	9,47	4,16
min FC	-9,72	-2,66	-2,89	-3,37	-5,89	-3,57	-3,24	-3,89	-3,31	-4,36
moyenne down	-2,60	-1,83	-2,00	-1,98	-2,28	-2,03	-1,82	-2,08	-2,02	-2,02
moyenne up	2,00	1,86	2,10	2,33	2,54	1,95	2,27	2,38	2,56	2,19
