# Supplementary material for: Modulation of miRNA Expression by Dietary Polyphenols in apoE Deficient Mice: A New Mechanism of the Action of Polyphenols
Source: PLoS One. 2012 Jan 10;7(1):e29837. doi: 10.1371/journal.pone.0029837 (PMC3254631; doi:10.1371/journal.pone.0029837)
Supplement: Table S4 — KEGG pathways of identified potential target genes of five commonly regulated miRNAs. (PDF) [file pone.0029837.s009.pdf]

Supplement table S4

	miR-291b-5p	miR-296-5p	miR-30c-1*	miR-467b*	miR-374*
1- and 2-Methylnaphthalene degradation	1	1	1	0	1
1,1,1-Trichloro-2,2-bis(4-chlorophenyl)ethane (DDT) degradation	0	0	1	0	0
2,4-Dichlorobenzoate degradation	0	0	2	0	0
ABC transporters - General	2	0	0	0	0
Adherens junction	2	5	2	0	0
Adipocytokine signaling pathway	2	4	1	3	0
Alanine and aspartate metabolism	1	1	2	1	1
Alkaloid biosynthesis II	0	1	2	0	0
Alzheimer's disease	1	1	2	0	0
Aminoacyl-tRNA biosynthesis	1	2	1	0	1
Aminophosphonate metabolism	0	0	1	0	0
Aminosugars metabolism	4	1	1	0	0
Androgen and estrogen metabolism	2	0	1	1	2
Antigen processing and presentation	4	1	2	0	2
Apoptosis	1	2	2	5	2
Arachidonic acid metabolism	5	3	5	1	0
Arginine and proline metabolism	4	0	3	0	1
Ascorbate and aldarate metabolism	2	0	0	1	2
ATP synthesis	0	0	2	0	1
Atrazine degradation	0	1	1	0	1
Axon guidance	4	6	7	1	0
B cell receptor signaling pathway	0	4	2	3	1
Basal transcription factors	1	0	1	3	1
Benzoate degradation via CoA ligation	1	2	2	2	2
Benzoate degradation via hydroxylation	0	0	1	0	1
beta-Alanine metabolism	3	0	2	1	0
Bile acid biosynthesis	2	0	1	1	3
Biosynthesis of steroids	1	0	0	1	0
Biotin metabolism	1	0	1	0	0
Bisphenol A degradation	3	1	1	1	3
Blood group glycolipid biosynthesis - neo-lactoseries	0	1	0	0	0
Butanoate metabolism	3	0	1	2	2
C21-Steroid hormone metabolism	2	1	0	0	1
Calcium signaling pathway	3	4	6	3	7
Caprolactam degradation	0	2	0	1	0
Carbon fixation	0	3	1	0	1
Cell adhesion molecules (CAMs)	10	4	1	0	2
Cell Communication	4	10	15	0	4
Cell cycle	6	4	5	4	3
Chondroitin sulfate biosynthesis	0	1	0	0	0
Circadian rhythm	1	1	1	0	1
Citrate cycle (TCA cycle)	1	0	0	1	0
Complement and coagulation cascades	5	4	1	1	1

Cyanoamino acid metabolism	0	1	1	0	0
Cysteine metabolism	1	2	1	1	0
Cytokine-cytokine receptor interaction	5	6	10	9	10
Diterpenoid biosynthesis	0	1	0	0	0
DNA polymerase	1	0	1	2	1
Dorso-ventral axis formation	1	3	3	0	1
ECM-receptor interaction	2	4	4	2	1
Ethylbenzene degradation	1	2	1	0	1
Fatty acid elongation in mitochondria	0	0	0	0	1
Fatty acid metabolism	2	0	0	2	1
Fc epsilon RI signaling pathway	2	5	3	2	3
Fluorene degradation	0	0	1	0	0
Focal adhesion	5	10	11	3	4
Folate biosynthesis	0	2	1	2	1
Fructose and mannose metabolism	3	1	4	1	4
Galactose metabolism	2	0	1	1	3
gamma-Hexachlorocyclohexane degradation	1	1	1	1	1
Gap junction	2	4	6	3	4
Glutamate metabolism	1	2	0	1	0
Glutathione metabolism	1	3	1	1	2
Glycan structures - biosynthesis 1	5	2	2	2	1
Glycan structures - biosynthesis 2	0	1	0	0	0
Glycan structures - degradation	0	1	1	0	0
Glycerolipid metabolism	0	3	1	0	1
Glycerophospholipid metabolism	3	7	3	1	2
Glycine, serine and threonine metabolism	3	0	3	1	3
Glycolysis / Gluconeogenesis	0	2	4	1	3
Glycosaminoglycan degradation	0	0	1	0	0
Glycosphingolipid metabolism	1	1	0	0	0
Glycosylphosphatidylinositol(GPI)-anchor biosynthesis	0	1	0	0	0
Glyoxylate and dicarboxylate metabolism	1	0	1	0	0
Hedgehog signaling pathway	0	3	1	1	0
Hematopoietic cell lineage	2	4	0	2	1
Heparan sulfate biosynthesis	0	1	2	0	0
Histidine metabolism	2	1	3	0	2
Huntington's disease	1	1	0	0	1
Inositol phosphate metabolism	1	3	2	3	1
Insulin signaling pathway	2	9	5	4	4
Jak-STAT signaling pathway	3	7	1	4	9
Keratan sulfate biosynthesis	1	0	0	0	0
Leukocyte transendothelial migration	2	4	0	1	4
Limonene and pinene degradation	1	2	1	1	1
Linoleic acid metabolism	4	1	1	3	2
Long-term depression	5	2	5	2	2
Long-term potentiation	1	3	3	1	0

Lysine biosynthesis	2	0	0	0	1
Lysine degradation	6	0	2	3	4
MAPK signaling pathway	14	15	6	3	5
Maturity onset diabetes of the young	1	0	1	1	0
Metabolism of xenobiotics by cytochrome P450	3	1	3	3	3
mTOR signaling pathway	2	2	1	1	1
Natural killer cell mediated cytotoxicity	1	5	9	4	6
Neuroactive ligand-receptor interaction	8	8	11	6	8
Neurodegenerative Disorders	0	1	1	0	1
N-Glycan biosynthesis	3	0	0	2	1
N-Glycan degradation	0	1	0	0	0
Nicotinate and nicotinamide metabolism	3	2	2	1	1
Nitrobenzene degradation	0	0	2	0	0
Nitrogen metabolism	0	1	1	1	2
Notch signaling pathway	0	2	2	0	0
Nucleotide sugars metabolism	3	0	0	1	2
O-Glycan biosynthesis	2	0	0	0	0
Olfactory transduction	0	1	0	0	1
One carbon pool by folate	0	1	0	0	2
Oxidative phosphorylation	2	3	3	2	3
Pantothenate and CoA biosynthesis	1	2	0	0	0
Parkinson's disease	1	2	0	0	0
Pentose and glucuronate interconversions	1	0	0	1	1
Pentose phosphate pathway	0	1	2	0	1
Phenylalanine metabolism	0	0	2	0	1
Phenylalanine, tyrosine and tryptophan biosynthesis	0	0	1	0	0
Phosphatidylinositol signaling system	1	5	3	5	2
Porphyrin and chlorophyll metabolism	1	0	0	1	0
Prion disease	0	1	1	0	0
Propanoate metabolism	1	1	0	3	4
Proteasome	1	1	4	0	0
Purine metabolism	6	3	8	6	0
Pyrimidine metabolism	6	2	2	3	1
Pyruvate metabolism	1	2	0	2	2
Regulation of actin cytoskeleton	6	10	13	2	3
Regulation of autophagy	0	1	0	0	3
Riboflavin metabolism	1	0	0	0	0
Ribosome	3	1	1	0	3
RNA polymerase	2	1	1	2	0
Selenoamino acid metabolism	0	1	2	0	0
SNARE interactions in vesicular transport	3	0	0	2	0
Starch and sucrose metabolism	1	2	0	3	2
Streptomycin biosynthesis	1	0	0	1	1
Styrene degradation	1	0	1	0	0
T cell receptor signaling pathway	1	4	3	3	2

Taste transduction	0	2	1	1	0
Taurine and hypotaurine metabolism	1	1	2	0	0
Terpenoid biosynthesis	1	0	0	1	0
Tetrachloroethene degradation	2	0	0	1	2
TGF-beta signaling pathway	2	3	3	3	1
Thiamine metabolism	1	0	0	0	0
Tight junction	2	3	4	0	0
Toll-like receptor signaling pathway	2	0	1	4	4
Tryptophan metabolism	2	0	4	2	1
Type I diabetes mellitus	5	0	0	2	0
Type II diabetes mellitus	1	4	1	1	1
Tyrosine metabolism	3	1	3	0	1
Ubiquinone biosynthesis	0	0	1	0	0
Urea cycle and metabolism of amino groups	1	1	2	0	0
Valine, leucine and isoleucine degradation	2	0	1	3	2
VEGF signaling pathway	5	3	3	1	1
Vitamin B6 metabolism	1	1	0	0	0
Wnt signaling pathway	2	7	4	0	0
