# Supplementary material for: Modulation of miRNA Expression by Dietary Polyphenols in apoE Deficient Mice: A New Mechanism of the Action of Polyphenols
Source: PLoS One. 2012 Jan 10;7(1):e29837. doi: 10.1371/journal.pone.0029837 (PMC3254631; doi:10.1371/journal.pone.0029837)
Supplement: Table S6 — Average fold-change and minimum and maximum values of fold-change of differentially expressed mRNAs. (PDF) [file pone.0029837.s011.pdf]

Supplement table S6

	ApoE -/-	Cafeic acid	Ferulic acid	Anthocyane	Catechin	Curcumin	Hesperidine	Naringin	Proanthocyanidin	Quercetin
average FC up-regulated	1,49	1,46	1,50	1,32	1,46	1,52	1,61	1,57	1,47	1,48
average FC down-regulated	0,73	0,72	0,70	0,77	0,72	0,71	0,68	0,70	0,68	0,71
max up	4,68	3,88	5,02	2,28	7,42	10,05	5,58	5,11	4,08	3,47
min down	0,38	0,30	0,29	0,27	0,20	0,32	0,38	0,40	0,35	0,20
