# Supplementary material for: Modulation of miRNA Expression by Dietary Polyphenols in apoE Deficient Mice: A New Mechanism of the Action of Polyphenols
Source: PLoS One. 2012 Jan 10;7(1):e29837. doi: 10.1371/journal.pone.0029837 (PMC3254631; doi:10.1371/journal.pone.0029837)
Supplement: Table S7 — KEGG pathways of differentially expressed mRNAs. (PDF) [file pone.0029837.s012.pdf]

Supplement table S7

pathways	ApoE -/-	Cafeic acid	Ferulic acid	Quercetin	Catechin	Hesperidin	Narangin	Proanthocyanidin	Anthocyanidin	Curcumin
1- and 2-Methylnaphthalene degradation	0	0	5	1	1	4	3	1	0	1
1,1,1-Trichloro-2,2-bis(4-chlorophenyl)ethane (DDT) degradation	0	0	0	0	0	0	0	0	0	0
2,4-Dichlorobenzoate degradation	0	0	1	0	0	2	0	0	0	0
ABC transporters - General	4	2	3	2	3	6	3	2	8	1
Acute myeloid leukemia	0	0	0	0	0	0	0	0	9	0
Adherens junction	3	2	9	4	1	14	1	4	13	3
Adipocytokine signaling pathway	3	2	6	7	2	10	1	3	5	6
Alanine and aspartate metabolism	0	1	3	1	1	4	1	1	4	1
Aldosterone-regulated sodium reabsorption	0	0	0	0	0	0	0	0	10	0
Alkaloid biosynthesis I	1	0	0	1	0	1	0	1	0	0
Alkaloid biosynthesis II	0	0	3	1	0	5	4	1	0	0
Alzheimer's disease	2	0	2	1	0	4	0	2	24	0
Amino sugar and nucleotide sugar metabolism	0	0	0	0	0	0	0	0	8	0
Aminoacyl-tRNA biosynthesis	2	3	4	3	1	3	1	1	5	1
Aminophosphonate metabolism	2	1	3	2	1	4	0	2	0	0
Aminosugars metabolism	2	1	7	4	4	7	2	2	0	1
Amyotrophic lateral sclerosis (ALS)	2	0	0	4	3	2	0	2	12	1
Androgen and estrogen metabolism	2	2	4	4	1	5	0	1	0	0
Antigen processing and presentation	9	2	19	13	4	18	3	5	10	15
Apoptosis	4	2	10	3	2	16	1	7	10	6
Arachidonic acid metabolism	4	1	11	5	0	20	2	5	11	0
Arginine and proline metabolism	4	4	7	3	2	6	5	4	7	0
Arrhythmogenic right ventricular cardiomyopathy (ARVC)	0	0	0	0	0	0	0	0	10	0
Ascorbate and aldarate metabolism	1	0	3	1	0	2	1	1	1	2
ATP synthesis	3	0	10	5	4	10	3	1	0	4
Atrazine degradation	0	0	1	0	0	0	0	0	0	0
Axon guidance	9	7	25	8	4	24	2	8	21	4
B cell receptor signaling pathway	2	3	10	1	1	12	1	4	10	6
Basal cell carcinoma	0	0	0	0	0	0	0	0	9	0
Basal transcription factors	3	2	2	3	1	6	0	1	6	1
Base excision repair	0	0	0	0	0	0	0	0	4	0
Benzoate degradation via CoA ligation	1	0	3	1	1	7	3	4	0	3
Benzoate degradation via hydroxylation	0	2	0	0	0	1	0	0	0	0
beta-Alanine metabolism	0	0	3	2	3	3	0	2	1	2
Bile acid biosynthesis	1	2	5	4	2	2	2	1	1	3
Biosynthesis of steroids	1	2	2	1	0	3	0	1	0	0
Biosynthesis of unsaturated fatty acids	0	0	0	0	0	0	0	0	7	0
Biotin metabolism	2	0	2	0	0	1	0	0	2	2
Bisphenol A degradation	2	0	4	1	0	3	2	1	0	3
Blood group glycolipid biosynthesis - lactoseries	0	1	1	2	0	3	0	0	0	0
Blood group glycolipid biosynthesis - neo-lactoseries	1	2	3	2	0	0	0	0	0	1
Butanoate metabolism	2	0	7	3	2	8	1	4	5	3
C21-Steroid hormone metabolism	1	2	2	1	0	2	0	0	0	1
C5-Branched dibasic acid metabolism	0	1	0	0	0	0	0	0	0	1
Calcium signaling pathway	13	9	27	10	5	29	8	10	33	8

Caprolactam degradation	0	0	2	2	1	2	2	2	0	1
Carbazole degradation	0	0	0	0	0	0	0	0	0	0
Carbon fixation	0	2	3	2	0	3	0	0	0	0
Cell adhesion molecules (CAMs)	15	8	24	16	4	30	5	8	18	15
Cell Communication	7	5	11	10	10	23	4	13	5	8
Cell cycle	2	3	18	5	2	26	3	9	13	7
Chemokine signaling pathway	0	0	0	0	0	0	0	0	22	0
Chondroitin sulfate biosynthesis	0	1	4	3	1	5	0	2	0	1
Circadian rhythm	1	2	1	1	0	1	0	1	0	0
Citrate cycle (TCA cycle)	3	4	3	1	0	6	2	0	5	2
Colorectal cancer	0	0	0	0	0	0	0	0	11	0
Complement and coagulation cascades	2	5	4	4	1	8	3	8	12	5
Cyanoamino acid metabolism	0	0	0	0	0	0	0	0	1	0
Cysteine and methionine metabolism	0	0	0	0	0	0	0	0	4	0
Cysteine metabolism	2	1	3	0	2	1	1	1	0	0
Cytokine-cytokine receptor interaction	15	13	52	11	9	33	13	15	21	11
Cytosolic DNA-sensing pathway	0	0	0	0	0	0	0	0	6	0
Dentatorubropallidoluysian atrophy (DRPLA)	1	1	1	0	1	1	2	2	0	0
D-Glutamine and D-glutamate metabolism	0	0	1	0	0	1	0	1	0	0
Diterpenoid biosynthesis	0	0	2	1	0	2	0	0	0	0
DNA polymerase	2	1	3	3	0	7	2	0	0	4
DNA replication	0	0	0	0	0	0	0	0	5	0
Dorso-ventral axis formation	2	2	6	4	2	5	1	0	2	3
ECM-receptor interaction	4	4	10	8	5	16	5	6	10	4
Endocytosis	0	0	0	0	0	0	0	0	21	0
ErbB signaling pathway	0	0	0	0	0	0	0	0	14	0
Ethylbenzene degradation	0	0	3	1	0	4	4	1	0	0
Fatty acid biosynthesis	0	2	0	0	0	2	0	0	2	0
Fatty acid elongation in mitochondria	0	1	0	0	0	2	1	0	1	1
Fatty acid metabolism	0	3	5	2	2	6	0	2	9	3
Fc epsilon RI signaling pathway	4	1	13	4	4	14	2	3	9	2
Fc gamma R-mediated phagocytosis	0	0	0	0	0	0	0	0	16	0
Fluorene degradation	0	0	0	0	0	0	0	0	0	0
Focal adhesion	11	5	18	15	10	45	4	11	27	4
Folate biosynthesis	1	1	5	1	4	9	1	3	0	2
Fructose and mannose metabolism	6	1	6	4	4	4	4	3	4	5
Galactose metabolism	4	0	3	3	3	4	2	3	2	6
gamma-Hexachlorocyclohexane degradation	1	0	4	0	1	7	1	3	0	3
Ganglioside biosynthesis	0	0	2	0	0	1	0	1	0	0
Gap junction	4	4	14	4	7	13	1	7	14	3
Globoside metabolism	2	0	2	1	1	2	1	1	0	2
Glutamate metabolism	1	4	6	3	1	5	1	4	0	1
Glutathione metabolism	3	3	6	5	0	11	2	8	6	3
Glycan structures - biosynthesis 1	3	4	17	9	3	20	4	5	0	4
Glycan structures - biosynthesis 2	4	3	6	6	1	13	1	2	0	5
Glycan structures - degradation	1	0	5	0	1	4	0	2	0	2
Glycerolipid metabolism	4	3	6	3	1	7	4	2	5	7
Glycerophospholipid metabolism	5	4	13	2	0	16	8	6	10	3

Glycine, serine and threonine metabolism	3	3	6	1	2	8	2	3	0	4
Glycolysis / Gluconeogenesis	5	2	6	3	1	5	2	2	7	3
Glycosaminoglycan degradation	1	0	5	0	1	3	0	2	3	1
Glycosphingolipid metabolism	3	2	4	4	0	8	1	3	0	5
Glycosylphosphatidylinositol(GPI)-anchor biosynthesis	0	2	3	1	0	3	0	1	3	1
Glyoxylate and dicarboxylate metabolism	0	1	3	2	1	1	1	0	3	0
GnRH signaling pathway	0	0	0	0	0	0	0	0	13	0
Hedgehog signaling pathway	2	2	5	4	0	9	2	4	3	1
Hematopoietic cell lineage	2	4	12	9	4	13	4	4	7	2
Heparan sulfate biosynthesis	0	1	2	3	1	1	1	0	0	0
Histidine metabolism	1	2	7	2	2	3	1	0	3	0
Huntington's disease	3	2	2	3	4	1	1	4	22	4
Inositol metabolism	0	1	0	0	0	0	0	1	0	0
Inositol phosphate metabolism	3	2	9	2		9	1	5	9	2
Insulin signaling pathway	9	6	19	11	4	32	5	12	16	9
Intestinal immune network for IgA production	0	0	0	0	0	0	0	0	3	0
Jak-STAT signaling pathway	10	9	20	9	6	25	8	12	15	11
Keratan sulfate biosynthesis	1	0	4	2	0	5	0	1	0	0
Leukocyte transendothelial migration	8	6	15	7	5	24	2	11	15	3
Limonene and pinene degradation	0	0	4	2	1	5	4	2	2	1
Linoleic acid metabolism	1	0	6	1	0	15	2	4	6	2
Long-term depression	2	2	11	1	4	15	2	3	13	4
Long-term potentiation	6	2	12	6	3	14	1	9	11	6
Lysine biosynthesis	0	1	0	0	0	0	0	1	0	0
Lysine degradation	4	2	8	5	1	6	1	5	5	4
Lysosome	0	0	0	0	0	0	0	0	17	0
MAPK signaling pathway	21	12	42	13	15	38	9	18	32	15
Maturity onset diabetes of the young	2	1	3	3	1	5	3	1	3	0
Metabolism of xenobiotics by cytochrome P450	3	3	8	4	0	20	2	9	7	3
Methane metabolism	0	0	1	0	0	1	1	0	1	1
Methionine metabolism	1	1	2	1	1	2	1	2	2	1
mTOR signaling pathway	2	2	5	6	1	11	2	5	8	2
Natural killer cell mediated cytotoxicity	5	3	14	6	3	15	1	8	15	7
Neuroactive ligand-receptor interaction	17	14	40	15	13	57	17	20	41	11
Neurodegenerative Disorders	4	0	1	3	3	5	1	4	0	3
Neurotrophin signaling pathway	0	0	0	0	0	0	0	0	16	0
N-Glycan biosynthesis	2	3	5	0	1	8	1	2	7	3
N-Glycan degradation	1	0	4	0	0	3	0	2	0	2
Nicotinate and nicotinamide metabolism	2	0	5	1	1	4	3	3	3	2
Nitrobenzene degradation	1	1	2	2	1	2	0	0	0	0
Nitrogen metabolism	3	2	3	1	2	2	0	3	4	0
Notch signaling pathway	2	3	6	5	0	8	2	5	7	1
Novobiocin biosynthesis	1	0	0	0	0	0	0	1	0	0
Nucleotide excision repair	0	0	0	0	0	0	0	0	6	0
Nucleotide sugars metabolism	1	0	2	1	0	2	1	1	0	2
O-Glycan biosynthesis	1	0	2	1	1	2	1	0	5	1
Olfactory transduction	1	0	6	2	5	2	1	5	16	2
One carbon pool by folate	0	0	2	2	1	1	1	2	0	1

Oxidative phosphorylation	8	4	18	10	11	23	4	10	9	5
p53 signaling pathway	0	0	0	0	0	0	0	0	9	0
Pantothenate and CoA biosynthesis	0	0	3	1	0	3	2	2	0	1
Parkinson's disease	0	0	5	0	1	4	1	1	13	2
Pentose and glucuronate interconversions	0	1	2	0	1	1	0	1	4	1
Pentose phosphate pathway	2	2	8	3	1	2	0	0	2	0
Peroxisome	0	0	0	0	0	0	0	0	11	0
Phenylalanine metabolism	2	2	5	1	1	4	4	2	2	0
Phenylalanine, tyrosine and tryptophan biosynthesis	1	0	1	0	0	1	0	1	0	1
Phosphatidylinositol signaling system	4	4	11	4	2	12	3	8	12	3
Prion disease	0	1	0	2	2	2	2	0	0	2
Propanoate metabolism	2	4	4	2	3	5	1	4	5	2
Proteasome	3	3	6	2	0	6	1	4	7	1
Protein export	1	0	3	1	0	0	0	0	4	0
Proximal tubule bicarbonate reclamation	0	0	0	0	0	0	0	0	3	0
Purine metabolism	5	9	25	14	6	26	6	5	18	7
Pyrimidine metabolism	4	4	14	8	3	17	4	2	15	6
Pyruvate metabolism	2	1	3	1	1	8	0	2	8	2
Reductive carboxylate cycle (CO2 fixation)	1	5	0	0	0	1	1	1	0	1
Regulation of actin cytoskeleton	11	6	20	14	8	44	5	14	33	6
Regulation of autophagy	2	1	4	1	0	7	2	0	2	2
Retinol metabolism	0	0	0	2	0	2	0	0	11	0
Riboflavin metabolism	1	0	3	1	1	3	1	0	0	1
Ribosome	2	6	5	1	3	12	2	4	14	4
RNA degradation - Mus musculus (mouse) (10)	0	0	0	0	0	0	0	0	10	0
RNA polymerase	0	1	5	2	2	5	1	0	3	0
Selenoamino acid metabolism	2	2	4	2	2	4	0	1	4	2
SNARE interactions in vesicular transport	2	0	4	0	2	5	1	1	4	3
Sphingolipid metabolism	0	0	0	0	0	0	0	0	6	0
Spliceosome	0	0	0	0	0	0	0	0	22	0
Starch and sucrose metabolism	2	2	8	3	4	10	2	2	4	2
Stilbene, coumarine and lignin biosynthesis	0	0	0	1	0	2	1	0	0	0
Streptomycin biosynthesis	0	0	0	0	1	2	1	0	0	0
Styrene degradation	0	1	0	0	0	0	0	0	0	1
Sulfur metabolism	1	0	1	0	0	3	0	0	1	0
Synthesis and degradation of ketone bodies	0	0	2	0	0	2	0	1	1	0
T cell receptor signaling pathway	3	3	13	4	2	16	2	7	14	9
Taste transduction	1	0	4	2	2	3	0	3	12	1
Taurine and hypotaurine metabolism	0	0	2	0	2	1	1	2	2	1
Terpenoid backbone biosynthesis	0	0	0	0	0	0	0	0	2	0
Terpenoid biosynthesis	0	1	0	0	0	2	0	1	0	0
Tetrachloroethene degradation	1	0	2	1	0	1	1	1	0	2
TGF-beta signaling pathway	6	3	11	4	3	20	3	6	7	7
Thiamine metabolism	1	0	1	0	1	0	0	0	1	0
Tight junction	10	4	14	4	5	20	3	12	16	5
Toll-like receptor signaling pathway	3	2	14	3	1	14	1	7	8	4
Tryptophan metabolism	2	2	12	5	4	11	3	5	8	3
Type I diabetes mellitus	9	5	19	10	2	17	4	3	8	12

Type II diabetes mellitus	3	2	9	6	1	11	2	4	5	2
Tyrosine metabolism	4	5	10	4	3	7	3	5	4	2
Ubiquinone biosynthesis	2	0	1	0	1	2	0	0	16	0
Urea cycle and metabolism of amino groups	1	0	3	0	1	3	3	4	0	0
Valine, leucine and isoleucine biosynthesis	2	1	3	2	1	1	1	0	2	0
Valine, leucine and isoleucine degradation	1	4	6	1	3	10	3	4	4	1
Vascular smooth muscle contraction	0	0	0	0	0	0	0	0	16	0
Vasopressin-regulated water reabsorption	0	0	0	0	0	0	0	0	6	0
VEGF signaling pathway	4	2	12	1	2	19	1	4	14	3
Vitamin B6 metabolism	1	0	1	0	1	0	0	0	0	0
Wnt signaling pathway	7	5	23	8	4	19	1	8	15	10
