# Supplementary material for: Modulation of miRNA Expression by Dietary Polyphenols in apoE Deficient Mice: A New Mechanism of the Action of Polyphenols
Source: PLoS One. 2012 Jan 10;7(1):e29837. doi: 10.1371/journal.pone.0029837 (PMC3254631; doi:10.1371/journal.pone.0029837)
Supplement: Table S8 — Common pathways of differentially expressed mRNAs in liver after supplementation with different polyphenols. (PDF) [file pone.0029837.s013.pdf]

Supplement table S8

Cell processes		ApoE -/-	Cafeic acid	Ferulic acid	Quercetin	Catechin	Hesperidin	Narangin	Proanthocyanidin	Anthocyanidin	Curcumin
	Adherens junction	3	2	9	4	1	14	1	4	13	3
	Antigen processing and presentation	9	2	19	13	4	18	3	5	10	15
	Axon guidance	9	7	25	8	4	24	2	8	21	4
	Cell adhesion molecules (CAMs)	15	8	24	16	4	30	5	8	18	15
	Cell Communication	7	5	11	10	10	23	4	13	5	8
	Cytokine-cytokine receptor interaction	15	13	52	11	9	33	13	15	21	11
	ECM-receptor interaction	4	4	10	8	5	16	5	6	10	4
	Focal adhesion	11	5	18	15	10	45	4	11	27	4
	Gap junction	4	4	14	4	7	13	1	7	14	3
	Leukocyte transendothelial migration	8	6	15	7	5	24	2	11	15	3
	Long-term depression	2	2	11	1	4	15	2	3	13	4
	Long-term potentiation	6	2	12	6	3	14	1	9	11	6
	Neuroactive ligand-receptor interaction	17	14	40	15	13	57	17	20	41	11
	Regulation of actin cytoskeleton	11	6	20	14	8	44	5	14	33	6
	Tight junction	10	4	14	4	5	20	3	12	16	5
Signalling											
	Adipocytokine signaling pathway	3	2	6	7	2	10	1	3	5	6
	B cell receptor signaling pathway	2	3	10	1	1	12	1	4	10	6
	Calcium signaling pathway	13	9	27	10	5	29	8	10	33	8
	Fc epsilon RI signaling pathway	4	1	13	4	4	14	2	3	9	2
	Insulin signaling pathway	9	6	19	11	4	32	5	12	16	9

	Jak-STAT signaling pathway	10	9	20	9	6	25	8	12	15	11
	MAPK signaling pathway	21	12	42	13	15	38	9	18	32	15
	mTOR signaling pathway	2	2	5	6	1	11	2	5	8	2
	Phosphatidylinositol signaling system	4	4	11	4	2	12	3	8	12	3
	Propanoate metabolism	2	4	4	2	3	5	1	4	5	2
	Purine metabolism	5	9	25	14	6	26	6	5	18	7
	Pyrimidine metabolism	4	4	14	8	3	17	4	2	15	6
	T cell receptor signaling pathway	3	3	13	4	2	16	2	7	14	9
	TGF-beta signaling pathway	6	3	11	4	3	20	3	6	7	7
	Toll-like receptor signaling pathway	3	2	14	3	1	14	1	7	8	4
	VEGF signaling pathway	4	2	12	1	2	19	1	4	14	3
	Wnt signaling pathway	7	5	23	8	4	19	1	8	15	10
Metabolism											
	ABC transporters - General	4	2	3	2	3	6	3	2	8	1
	Bile acid biosynthesis	1	2	5	4	2	2	2	1	1	3
	Fructose and mannose metabolism	6	1	6	4	4	4	4	3	4	5
	Glycerolipid metabolism	4	3	6	3	1	7	4	2	5	7
	Glycolysis / Gluconeogenesis	5	2	6	3	1	5	2	2	7	3
	Lysine degradation	4	2	8	5	1	6	1	5	5	4
	Methionine metabolism	1	1	2	1	1	2	1	2	2	1
	Oxidative phosphorylation	8	4	18	10	11	23	4	10	9	5
	Starch and sucrose metabolism	2	2	8	3	4	10	2	2	4	2
	Tryptophan metabolism	2	2	12	5	4	11	3	5	8	3
	Type I diabetes mellitus	9	5	19	10	2	17	4	3	8	12
	Type II diabetes mellitus	3	2	9	6	1	11	2	4	5	2
	Tyrosine metabolism	4	5	10	4	3	7	3	5	4	2

	Valine, leucine and isoleucine degradation	1	4	6	1	3	10	3	4	4	1
Other											
	Ribosome	2	6	5	1	3	12	2	4	14	4
	Natural killer cell mediated cytotoxicity	5	3	14	6	3	15	1	8	15	7
	Huntington's disease	3	2	2	3	4	1	1	4	22	4
	Hematopoietic cell lineage	2	4	12	9	4	13	4	4	7	2
	Complement and coagulation cascades	2	5	4	4	1	8	3	8	12	5
	Cell cycle	2	3	18	5	2	26	3	9	13	7
	Apoptosis	4	2	10	3	2	16	1	7	10	6
	Aminoacyl-tRNA biosynthesis	2	3	4	3	1	3	1	1	5	1
